# Roles of physical disturbance and biome properties in shaping microbial communities within Indian Ocean eddies

**DOI:** 10.1093/ismeco/ycaf110

**Published:** 2025-07-02

**Authors:** Melissa L Brock, Alyse A Larkin, Adam C Martiny

**Affiliations:** Department of Ecology and Evolutionary Biology, University of California, Irvine, CA 92697, United States; Global Ocean Monitoring and Observing, National Oceanic and Atmospheric Administration, Silver Spring, MD 20910, United States; Department of Earth System Science, University of California, Irvine, CA 92697, United States; Department of Ecology and Evolutionary Biology, University of California, Irvine, CA 92697, United States; Department of Earth System Science, University of California, Irvine, CA 92697, United States

**Keywords:** microbiome, community diversity, functional diversity, taxonomic composition, disturbance, dispersal, environmental selection, cyclonic eddy, anticyclonic eddy

## Abstract

Oceanic eddies create localized upwelling and downwelling systems and are thought to alter microbial communities through environmental selection and dispersal. Though how these eddy-driven mechanisms contribute to microbial outcomes within a broader environmental context is unknown. We proposed that (1) eddies are a large disturbance that exert a significant influence on bacterial community and functional diversity as well as taxonomic and functional composition and (2) that the combined processes of environmental selection and dispersal determine bacterial outcomes within eddies. To address these hypotheses, we integrated bacterial genomics and environmental conditions from 26 eddies across the Indian Ocean. We observed that the biome had a strong, primary influence in shaping all aspects of bacterial communities with eddies playing a weak, secondary role. Additionally, there was minimal evidence of an effect of environmental selection or dispersal in shaping bacterial community diversity. Our observations highlight the variability in bacterial responses within and between eddy types and emphasize the importance of understanding eddy characteristics and broader biome attributes in interpreting bacterial responses.

## Introduction

Oceanic eddies are commonly occurring mesoscale features (10–200 km) that alter marine ecosystems. Eddies are circular currents of water that are broadly categorized by their polarity as cyclones or anticyclones. Within cyclones, surface divergence results in the doming of isopycnals, bringing colder, more nutrient-rich waters to the surface (i.e. localized upwelling). In contrast, within anticyclones, surface convergence results in a deepening of the isopycnals, bringing warmer, more nutrient-depleted waters and organic matter to depth (i.e. localized downwelling). Although these features are mesoscale and cause localized effects, they are ubiquitous features across the world’s oceans [1–3], increasing the temporal and spatial extent of eddy impacts on bacterial communities. This warrants a synthetic view of eddy influence on marine bacterial community diversity, composition, and function.

Eddies are physical disturbances whose impact on bacterial communities can be viewed through two frameworks. In the first framework, eddies are a selective force whose physical, vertical transport of water masses with differing geochemical compositions changes the environment, resulting in a different ecosystem than the surrounding waters. This environmental change selects for phytoplankton communities in the surface ocean in a predictable way. Through enhanced nutrient input, cyclones promote the growth of larger eukaryotic phytoplankton, such as diatoms and haptophytes [4, 5], whereas anticyclones are oligotrophic structures that favor the growth of *Prochlorococcus* [6] and nitrogen-fixing cyanobacteria [7]. These changes in the phytoplankton community have cascading effects on the heterotrophic community through facilitation. Phytoplankton release of dissolved organic matter, particularly carbon (DOC), regulates heterotrophic activity in the surface ocean [8, 9]. As such, shifts in the type and lability of DOC can alter the heterotrophic community. For example, during active downwelling within anticyclones, mortality of nano- and micro-phytoplankton increases, resulting in higher DOC concentrations and leading to increased heterotrophic bacterial abundance [10]. Additionally, within anticyclones, low nucleic acid cells, which are k-strategist cell types with smaller, less flexible genomes, thrive due to their oligotrophic lifestyles [11]. In contrast, during active upwelling within cyclones, heterotrophic bacterial abundances were increased due to increases in inorganic nutrient concentrations [10]. Furthermore, cyclones have been observed to favor the increase of high nucleic acid cells, which are r-strategist cell types with larger, more flexible genomes that can exploit nutrient pulses; this boom-and-bust cycle reshapes communities, leading to different community compositions within anticyclones and cyclones [11]. Thus, within the first framework, a sequence of events unfolds, where physical displacement of water masses can trigger changes in the geochemical environment, subsequently selecting for specific phytoplankton communities which can alter the heterotrophic community.

In the second framework, dispersal determines bacterial community outcomes. Within this framework, eddies are disturbances that disperse microbes laterally and vertically. Eddies can entrain microbial communities from their original water masses, transporting them laterally into different biomes, where these communities can persist for months [12]. Additionally, vertical displacement of mesopelagic and deep-water bacteria into the surface ocean can occur within cyclones [13–15], whereas vertical displacement within anticyclones can increase the depth distribution of surface ocean bacteria into the mesopelagic [16]. Thus, the dispersal of microbial communities through entrainment or vertical transport can lead to the establishment of new species, resulting in community turnover and changes in diversity. Eddy-driven dispersal can also lead to variation in biogeochemical function. For example, an eddy in the eastern tropical South Pacific laterally transported a sulfide-oxidizing denitrifying bacteria from sulfidic shelf waters to sulfide-free offshore waters where it continued to perform cryptic sulfur-cycling [17]. Lastly, the extent of dispersal within an eddy is likely influenced by the physical properties of that eddy, including polarity, point of origin, and vertical intensity, making these important variables in understanding the resulting bacterial communities.

Currently, the role of eddy-driven environmental selection and dispersal in shaping bacterial communities is unresolved, specifically within a broader environmental context. Here, we test these ideas in the Indian Ocean because of its numerous eddy fields across different biomes [18–20]. We asked: (1) What are the relative roles of biome versus eddy presence in shaping bacterial diversity? and (2) What taxonomic and functional changes are occurring within eddies and are these changes consistent across eddy type and biome? (1) We proposed that eddies would be the primary influence shaping bacterial community diversity and functional diversity through the combined processes of environmental selection and dispersal and that the biome would have a secondary effect. (2) We expected that community composition and function would be similar across eddies of the same polarity within environmentally similar biomes.

## Materials and methods

### Field sampling and eddy identification

Environmental conditions of temperature, nutrient concentrations (nitrate, nitrite, phosphate, and silicate), and nutricline depth were collected along two repeat hydrography GO-SHIP transects at predefined stations in the western (I07N) and eastern (I09N) Indian Ocean and were analyzed as previously described [21]. A complete description of sample collection and processing is also provided in Supplemental Materials and Methods. Nutrient stress indices derived from *Prochlorococcus* genomes were obtained from Ustick *et al*. [22]. Eddies that intersected the transects were identified and characterized using AVISO’s Mesoscale Eddy Trajectory Atlas (META3.2exp NRT). Eddy detection was based on absolute dynamic topography which was calculated as the sum of mean dynamic topography and sea level anomalies [3]. All samples within eddies were selected for analysis, resulting in one to nine samples per anticyclonic eddy and one to eight samples per cyclonic eddy (Table 1). Additionally, two samples north of each eddy and two samples south of each eddy were selected as reference samples, for a total of four reference samples per eddy. For each eddy sample, the distance to the center of the eddy was calculated. Samples that were $\le$25 km away from the center point were categorized as being in the center of the eddy, while samples that were $>$25 km away were categorized as being on the edge of the eddy.

**Table 1:** Physical attributes of eddies at the time of sampling.

Eddy ID	Polarity	Biome	Dates of sample collection (DD month YYYY)	Number of samples	Sample positions in eddy^a^	Age (days)	Amplitude (m)	Area (km^2^)	Radius (km)	Speed (m/s)
Cyc1	Cyclone	Eastern Gyre	22 March 2016–23 March 2016	2	E, E	350	0.157	36 100	115.35	0.2925
Cyc2	Cyclone	Eastern Gyre	24 March 2016	2	E, E	280	0.1591	29 800	107.85	0.361
Anticyc1	Anticyclone	Eastern Gyre	24 March 2016	2	E, C	17	0.0525	26 600	109.35	0.1587
Cyc3	Cyclone	Eastern Gyre	25 March 2016	1	E	16	0.0569	9140	64.8	0.2804
Cyc4	Cyclone	Eastern Gyre	27 March 2016	4	E, E, E, E	165–166	0.0831–0.0918	27 000–29 000	98.8–100.4	0.2643–0.2717
Cyc5	Cyclone	Eastern Gyre	28 March 2016	2	E, E	51–52	0.0451–0.0501	14 300–15 500	70–75.5	0.2022–0.2112
Anticyc2	Anticyclone	Eastern Gyre	29 March 2016	1	E	13	0.0213	11 600	62.5	0.1251
Cyc6	Cyclone	Eastern Gyre	30 March 2016	6	E, E, C, C, E, E	120–121	0.0147–0.0163	19 700–20 000	83.3–83.95	0.0992–0.097
Anticyc3	Anticyclone	Eastern Gyre	31 March 2016	4	E, E, C, E	192	0.0187	13 100	65.6	0.1377
Cyc7	Cyclone	Eastern Equatorial	11 April 2016–12 April 2016	5	E, E, C, C, E	NA	0.0068	14 600	72.2	0.1351
Anticyc4	Anticyclone	Eastern Equatorial	13 April 2016–14 April 2016	9	E, E, E, E, E, C, E, E, E	23–24	0.0248–0.0251	41 500–45 200	120.8–126.3	0.3775–0.3806
Cyc8	Cyclone	Bay of Bengal	14 April 2016–15 April 2016	7	E, E, E, E, E, E, E	14	0.0307	62 800	166.1	0.3393
Anticyc5	Anticyclone	Bay of Bengal	16 April 2016	5	E, E, E, E, E	74	0.0128	24 600	94.8	0.1673
Cyc9 (E–W)^b^	Cyclone	Bay of Bengal	17 April 2016–18 April 2016	6	E, E, E, E, E, E	79–80	0.0537–0.0566	47 000–48 700	137.8–138.45	0.2515–0.255
Cyc9 (S–N)^c^	Cyclone	Bay of Bengal	20 April 2016–21 April 2016	8	E, E, E, E, E, E, E, E	82–83	0.0543–0.055	44 900–47 900	126.25–134	0.2566–0.2598
Anticyc6	Anticyclone	Bay of Bengal	21 April 2016–22 April 2016	5	E, E, E, E, E	7–8	0.0306–0.0385	42 000–52 100	118.6–132.1	0.1639–0.1866
Cyc11	Cyclone	Bay of Bengal	23 April 2016	5	E, E, E, E, E	61–62	0.0481–0.0544	33 900	129.8–145.15	0.2014–0.2081
Cyc12	Cyclone	Western Gyre	27 April 2018	2	E, E	35	0.0291	7300	56.1	0.1587
Anticyc7	Anticyclone	Western Gyre	01 May 2018	1	E	38	0.0398	13 700	81.25	0.2193
Anticyc8	Anticyclone	Western Gyre	02 May 2018–03 May 2018	6	E, E, E, E, E, E	17	0.0702	25 900	119.65	0.3135
Anticyc9	Anticyclone	Western Gyre	04 May 2018	1	E	70	0.02	4210	44.9	0.207
Anticyc10	Anticyclone	Western Gyre	05 May 2018–06 May 2018	6	E, E, E, E, E, E	24–25	0.0449–0.0522	34 700–37 400	110.35–113.9	0.2557–0.2645
Anticyc11	Anticyclone	Western Gyre	07 May 2018–08 May 2018	2	C, C	18	0.0164	15 000	70.35	0.1262
Cyc13	Cyclone	Western Equatorial	12 May 2018–13 May 2018	5	E, E, E, E, E	37	0.0502	56 900	146.5	0.3216
Cyc14	Cyclone	Western Equatorial	14 May 2018–15 May 2018	2	E, E	9	0.0054	21 200	89.9	0.1345
Cyc15	Cyclone	Western Equatorial	22 May 2018–23 May 2018	6	E, E, E, C, C, C	0–1	0.0055–0.0073	10 100–20 000	59.25–82.65	0.1914–0.2125
Anticyc12	Anticyclone	Arabian Sea	01 June 2018–02 June 2018	8	E, E, E, E, E, E, E, E	11–12	0.1022–0.1054	71 800	163.4–168.7	0.2965–0.2993

a Positions of each sample within an eddy listed south to north where E indicates edge and C indicates center. For example, in Anticyc 1 which contains 2 samples, “E, C” indicates that the southernmost sample is in the edge of the eddy and that the northernmost sample is in the center of the eddy.

b Longitudinal sampling of cyclone 9 in the Bay of Bengal occurred from east to west.

c Latitudinal sampling of cyclone 9 in the Bay of Bengal occurred from south to north.

### Sequencing

Microbial DNA was collected from surface depths (2–7 m) every 4–6 h along a western (GO-SHIP I07N) and eastern (GO-SHIP I09N) transect in the Indian Ocean. DNA was extracted and sequenced for 16S rRNA amplicons (I07N and I09N), *rpo*C1 amplicons (I09N), and short-read metagenome sequences (I07N and I09N). A complete description of the library preparation and sequencing methods are provided in Supplemental Materials and Methods. Briefly, 16S rRNA sequences were obtained from NCBI’s Sequence Read Archive (SRA) (PRJNA656268) and were generated through amplification of the V4–V5 region using the 515F-C and 926R primer set [21, 23]. *rpo*C1 sequences were obtained from SRA (PRJNA522445) and were generated using cyanobacteria-specific primers 5M_newF and SAC1039R [24] with Illumina-specific Nextera transposase adapters [25]. Short-read metagenome sequences were obtained from SRA (PRJNA656268) and were generated through tagmentation and custom Nextera DNA-style 8-bp unique dual index barcodes [26].

### Bioinformatics

Bioinformatics methods for all three types of sequencing are summarized in Fig. 1 of the Supplemental Materials and Methods. 16S rRNA and *rpo*C1 sequences were processed as previously described [21, 25], and a complete description of these methods is also provided in Supplemental Materials and Methods. For short-read metagenome sequences, adapters were removed using trimmomatic v0.39 [27] with a maximum allowed mismatch of 2 bp, a palindrome clip threshold of 30 bp, and a simple clip threshold of 10 bp. Trimmomatic was used for initial quality control through sliding window trimming with a window size of 4 bp and average quality threshold of 15. Reads that were less than 36 bp in length were discarded. PhiX sequences were removed using BBDuk with a hamming distance of 1 and kmer size of 31. Additional quality control was performed on paired and unpaired reads using ngless [28]. The longest substring meeting a minimum quality threshold of 25 for all bases was retained from each read. Reads that were <45 bp in length were discarded, and unpaired reads were retained. Using ngless and bwa [29], quality-controlled reads were mapped to the marine reference database (95% redundancy; excluding rare genes) from the Global Microbial Gene Catalog (GMGC) [30], and all found alignments for paired and unpaired reads were output as unigenes (i.e. representative genes). Unigenes were normalized using FPKM and were functionally annotated by GMGC using eggNOG mapper [31].

### Data analysis

All analyses were performed in R (version 4.2.3) [32]. Environmental variables of temperature, nutricline depth, and nutrient concentrations (nitrate, nitrite, phosphate, and silicate) were centered and scaled (“scale”; R base). Principal component analysis (PCA) (“ordinate”; phyloseq) [33] and a permutational ANOVA (“adonis2”; permutations = 999, method = “euc”, vegan) [34] was performed to determine how the environmental context varied.

16S rRNA reads were normalized to 10 000 sequences per sample using rarefaction (“rrarefy”; vegan), and singletons were removed. Normalization depth was selected based on the minimum library size of quality-controlled samples (10 299 reads) [21] in order to retain all samples in subsequent analyses. The Shannon Index (“diversity”; vegan) was used to calculate compositional diversity from 99% OTUs and was used to calculate functional diversity from unigenes. Correlations between functional and community diversity were performed (“cor.test”; method = “pearson”; R stats). A two-sided *t*-test was performed to determine if alpha-diversity varied between the western and eastern transects (“t.test”; R stats). To determine if alpha-diversity changed within eddies, Δ Shannon was calculated as the Shannon Index of each eddy sample minus the average Shannon Index of the nearest non-eddy samples. Changes in environmental conditions were also calculated using this method.

Random forest regression (RFR) was used to determine the relative roles of biome properties versus eddy type in predicting alpha-diversity. For all RFR models, the models were trained on 70% of the data, used a *k*-fold cross-validation of 10, and were iteratively optimized. For community diversity, the final model parameters were mtry = 2, nodesize = 5, maxnodes = 29, and ntree = 550 (“train”; caret) [35]. A second model using eddy samples was constructed to identify which eddy properties were good predictors of community diversity with final model parameters of mtry = 5, nodesize = 10, maxnodes = 17, and ntree = 300. For functional diversity of unigenes, the final model parameters were mtry = 1, nodesize = 10, maxnodes = 21, and ntree = 300. For all RFR models, node purity and increase in mean squared error were used to identify the best predictors. Correlations of alpha-diversity with the best predictors were performed.

Partial mantel tests (permutations = 9999, method = “pearson”, “mantel.partial”; vegan) were performed on large eddies with adequate sample sizes (*n* ≥ 7 eddy samples; *n* = 4 non-eddy samples) to identify correlations between environmental variation and geographic distance with changes in community structure. A Euclidean environmental matrix (“dist”; R stats) was constructed using centered and scaled temperature, salinity, nutricline depth, silicate concentrations, nitrite concentrations, P-stress, and Fe-stress. A geographic distance matrix was constructed from longitude and latitude using Haversine great circle distance (“distm”; geosphere) [36]. A Bray–Curtis dissimilarity matrix was constructed from the rarefied 99% OTU table (“vegdist”; vegan).

Taxa were assigned using RDP’s Naïve Bayesian classifier and the SILVA138 reference database with a minimum bootstrap confidence of 80 (“assignTaxonomy”; dada2) [37]. Differential abundance analysis of genera by eddy type was performed (“DESeq”; DESeq2) [38]. To examine broad-scale patterns in taxonomic composition, genera in ≥1% relative abundance within one or more samples were categorized as dominant taxa. A two-way ANOVA was performed to determine if the total abundance of dominant taxa varied by biome or eddy type (“aov”; R stats), and then, a one-way ANOVA was performed to determine if it varied by eddy type for each transect. Similarly, to examine broad-scale patterns in functional composition, unigene abundance tables were aggregated to the COG functional category level [39] by summing normalized unigene relative abundances based on their COG category annotations that were output by GMGC. Two-way and one-way ANOVAs were performed to identify significant differences in COG category abundances as described above.

Differential abundance of unigenes by eddy type was determined through pairwise comparisons. Log-linear models were constructed for each unigene for each pairwise comparison using a coefficient of interest of 2, and permutation tests were performed to calculate *P*-values of each log-linear model using 1000 bootstraps (“fitLogNormal”; metagenomeSeq) [40]. *P*-Values were corrected using the Benjamini–Hochberg correction method (“p.adjust”; R stats). Unigenes with adjusted *P*-values <.05 were designated as differentially abundant. For each pairwise comparison, differentially abundant unigenes that had functional annotations were centered and scaled, and heatmaps were constructed (“Heatmap”; ComplexHeatmap) [41].

A subset of the differentially abundant unigenes had eggNOG text annotations that are metabolically or biologically important (Table S1). For each subset, unigenes that were found in ≥25% of samples (*n* = 50) were selected, centered, and scaled. PCA was performed for each subset, and correlations of environmental and eddy parameters with PC1 and PC2 were performed.

## Results

### Eddy locations and descriptions

**Figure 1:**
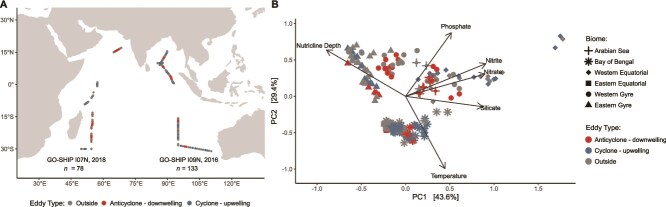
Geographic distribution and environmental context of eddies across the Indian Ocean. (**A**) Eddies identified through AVISO’s Mesoscale Eddy Trajectory Atlas (META3.2exp NRT) had an unequal distribution of polarities across the biomes. (**B**) Principal component analysis (PCA) revealed that environmental conditions are primarily structured by the biome.

The distribution and characteristics of eddies (*n* = 26) differed across Indian Ocean biomes (Fig. 1, Table 1). Here, the Indian Ocean was divided into six broad biomes: the western and eastern gyres, the western and eastern equatorial regions, the Arabian Sea, and the Bay of Bengal. The gyre regions, which extend from 30°S to 12°S, are oligotrophic and have cooler temperatures, while the equatorial regions, which extend from 12°S to 5°N, are warmer and have shallower nutricline depths (i.e. higher levels of nutrient supply). The Arabian Sea, which extends from 5°N to 18°N in the western Indian Ocean, is warm with shallow nutricline depths, and the Bay of Bengal, which extends from 5°N to 18°N in the eastern Indian Ocean, is warm, has shallow nutricline depths, and is P-stressed. Although the total number of eddies was similar in the western (*n* = 11) and eastern Indian Ocean (*n* = 15), eddy types were unevenly distributed across biomes from the same latitude. For example, the western gyre was dominated by anticyclones (*n* = 5), whereas the eastern gyre was dominated by cyclones (*n* = 6). Additionally, within the northern Indian Ocean, only one anticyclone was found in the Arabian Sea, whereas a mixture of cyclones (*n* = 2) and anticyclones (*n* = 2) were spread throughout the Bay of Bengal. Across the biomes, eddies varied widely in age (0–350 days), amplitude (0.005–0.159 m), radius (44.9–168.7 km), and speed (0.097–0.381 m/s) (Table 1). Overall, we observed that certain eddy types were more predominant within specific biomes and that there were notable differences in their physical attributes.

### Impact of eddies on environmental conditions

Environmental conditions were mainly linked to the biome with eddy polarity altering environmental conditions in an inconsistent way. Each region had a significantly different environmental context (PERMANOVA adj. *P* = .001) (Fig. 1) upon which eddies had a variable influence (PERMANOVA adj. *P* > .05). Although cyclones are expected to decrease temperature and increase nutrient supply to the surface ocean, this was observed in <50% of the cyclones (Fig. S1). Conversely, anticyclones are expected to vary minimally in temperature and are expected to decrease nutrient supply, which was more commonly observed (75% of anticyclones) (Fig. S1). Small increases in nutrient concentrations were observed within some cyclonic and anticyclonic eddies. Increases in nitrate and nitrite concentrations were rare, while increases in phosphate and silicate concentrations occurred more frequently (Fig. S2). Although changes in nutrient concentrations were small, fluctuations occurred in the type of nutrient stress that the microbial communities experienced. The majority of the Indian Ocean experienced N-stress, but cyclones and anticyclones sometimes experienced a P-stressed environment because of the overarching conditions in the Bay of Bengal (Fig. S3). Thus, we observed that although most fluctuations in nutrient concentrations within eddies were small, changes in nutrient supply frequently occurred.

### Impact of eddies on bacterial community diversity

**Figure 2:**
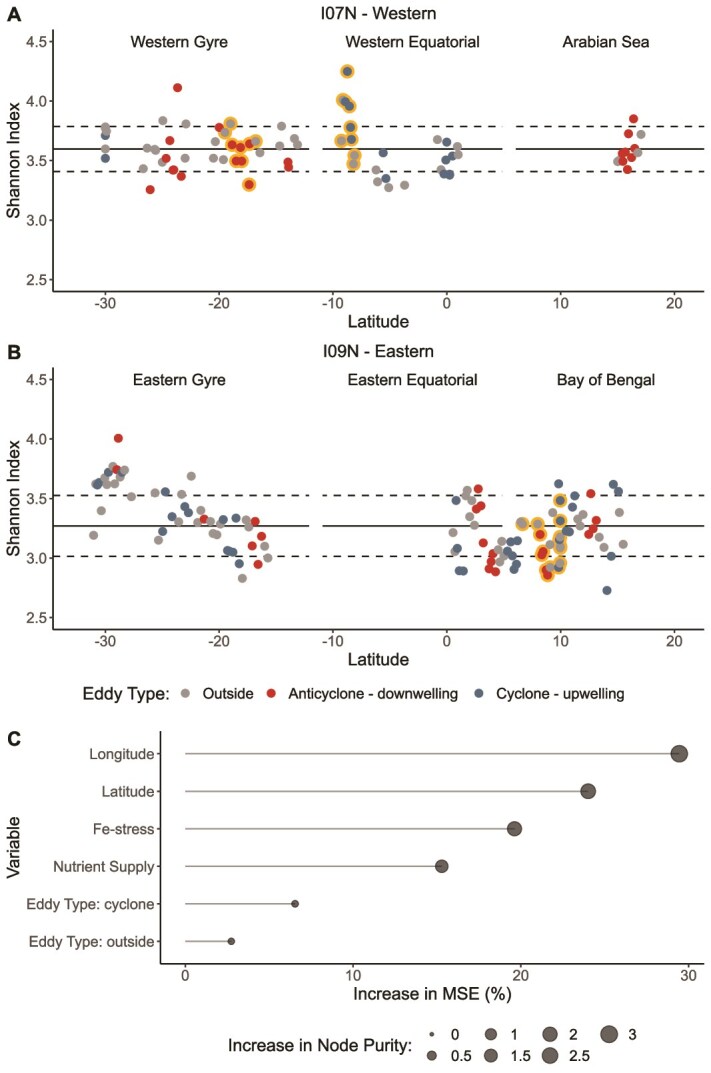
Spatial trends and best predictors of bacterial community diversity. (**A** and **B**) Longitudinal and latitudinal trends in community diversity calculated using the Shannon Index. Highlighted points indicate samples within eddies that are discussed in detail within the text. (**C**) Best predictors of community diversity as determined through random forest regression.

**Figure 3:**
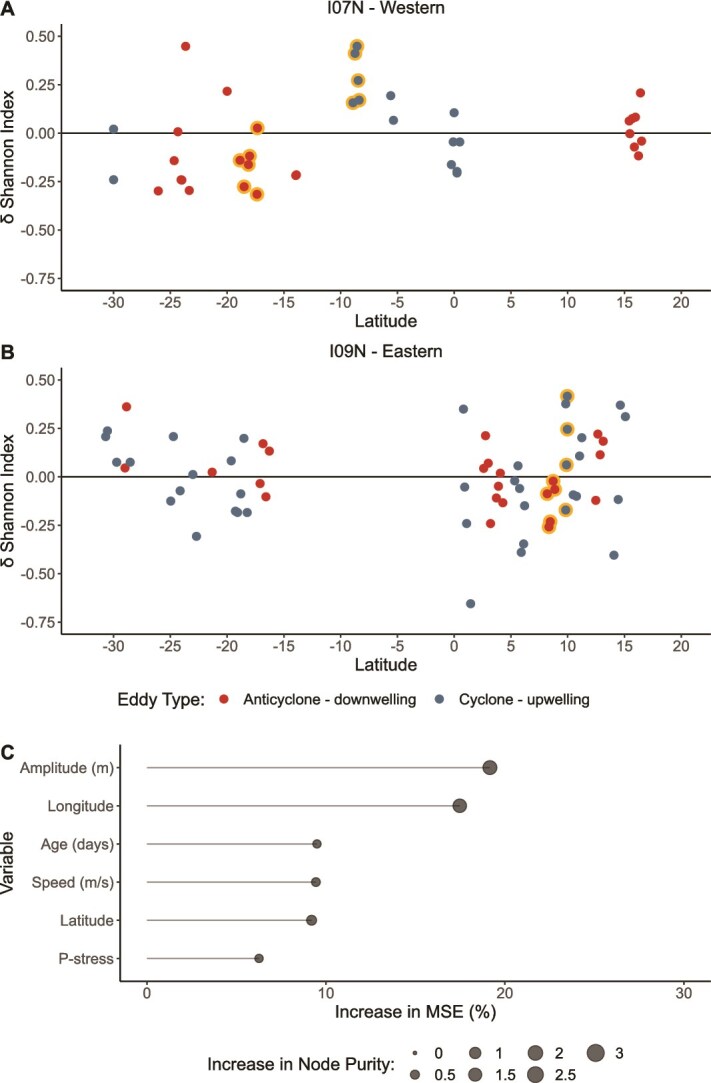
Changes in bacterial community diversity within eddies. (**A** and **B**). Changes in community diversity within each eddy as compared to the average value of the nearest neighbor control samples. Highlighted points indicate samples within eddies that are discussed in detail within the text. (**C**) Eddy attributes that are the best predictors of community diversity as determined through random forest regression.

The primary factor shaping bacterial community diversity was the biome with eddies playing a secondary role. Spatial trends in the Shannon Index showed that diversity was significantly higher (*P* < .001) in the western Indian Ocean compared to the eastern and that there was a strong latitudinal gradient in the eastern gyre (Fig. 2). Random forest regression (RFR) identified longitude and latitude as the most important variables for model accuracy followed by Fe-stress, nutrient supply, and eddy type (Fig. 2), indicating that biome properties are the primary factors shaping community diversity. This RFR model was able to closely estimate the Shannon Index and explained 49% of the variance (Fig. S4). Correlation analysis showed that latitude correlated negatively with community diversity (*r* = −0.37, *P* < .0001), while Fe-stress (*r* = 0.43, *P* < .0001) had a positive correlation, and nutrient supply had a weak, nonsignificant positive correlation (*r* = 0.11, *P* = .13). Although eddy type contributed a small amount to model accuracy in the initial RFR, eddies did alter community diversity with both cyclones and anticyclones showing decreases and increases in community diversity across the biomes (Fig. 3). RFR, using only eddy samples, identified eddy location, amplitude, age, and speed as important variables for model accuracy (Fig. 3). This model was able to effectively predict the Shannon Index and explained 59% of the variance (Fig. S4). Correlation analysis showed that eddy age negatively correlated with community diversity (*r* = −0.28, *P* = .008), while amplitude had a positive correlation (*r* = 0.40, *P* = .0001), and speed had a weak, nonsignificant positive correlation (*r* = 0.19, *P* = .08). Therefore, when eddies are younger, have higher amplitudes and faster speeds, community diversity may increase within the eddy. This was best exhibited in two places: (1) a 37-day old cyclone in the western equatorial region at 9°S and (2) the longitudinal transect through an 80-day old cyclone in the Bay of Bengal at 10°N (Table 1; Fig. 3). The opposite trend in community diversity (i.e. −Δ diversity) was observed in two anticyclones: (1) a 24-day old anticyclone in the western gyre at 18°S and (2) a 74-day old anticyclone in the Bay of Bengal at 8°N (Table 1; Fig. 3). This opposing response highlights the contrasting roles that eddy polarity may play in shaping community diversity. Along the western transect, cyclones with a +Δ diversity corresponded with shallower nutriclines and higher silicate concentrations, while cyclones with a −Δ diversity corresponded with deeper nutriclines and lower silicate concentrations (Fig. S5). However, this pattern was not observed in cyclones along the eastern transect, and no discernible relationship between changes in community diversity and environmental conditions could be identified along either transect for anticyclones (Fig. S5). Therefore, we observed that the biome is the most important predictor of bacterial community diversity with eddy characteristics of age, amplitude, and speed being important secondary predictors, although the exact relationships between these eddy characteristics, environmental conditions, and changes in bacterial community diversity are unresolved.

### Impact of eddies on bacterial functional diversity

The primary factor shaping functional diversity was the biome with a limited secondary role of eddies. Similar to what was observed in community diversity, spatial trends in the Shannon Index showed that functional diversity of unigenes was significantly higher (*P* < .001) in the western Indian Ocean compared to the eastern (Fig. S6). However, unlike community diversity, there were no obvious latitudinal trends in functional diversity. RFR revealed that geographic location was the most important variable for model accuracy, followed by Fe-stress, P-stress, and nutrient supply (Fig. S6), indicating that properties of the biome (e.g. location and nutrient conditions) are the primary factors shaping functional diversity of unigenes. Using these variables, the model was able to closely estimate the Shannon Index and explained 41.4% of the variance (Fig. S6). Latitude (*r* = −0.36, *P* < .0001) and P-stress (*r* = −0.39, *P* < .0001) had significant negative relationships with functional diversity, while Fe-stress had a significant positive relationship (*r* = 0.40, *P* < .0001). Nutrient supply had a positive but nonsignificant relationship with functional diversity (*r* = 0.14, *P* = .053). When eddy type was included in the model, it decreased model accuracy and reduced the variance explained. Although eddy type was not a good predictor of functional diversity, functional diversity within eddies differed from non-eddy samples. Both cyclones and anticyclones had decreases and increases in functional diversity across the biomes (Fig. S7). These changes in functional diversity significantly correlated with changes in community diversity along the western transect (*r* = 0.504, *P* = .001) but not the eastern (*r* = 0.238, *P* = .067). Additionally, changes in functional diversity significantly correlated with nitrate concentrations (*r* = 0.43, *P* < .001) and changes in nutrient supply (*r* = −0.28, *P* = .02) along the eastern transect but not the western. A notable pattern from Δ community diversity was repeated in Δ functional diversity. In the two previously described cyclones where community diversity increased, the majority of samples within these eddies also exhibited an increase in functional diversity (Fig. S7). These specific cyclones concurrently experienced increases in nutrient supply and nitrate concentrations (Figs. S1 and S2). Conversely, within the two previously described anticyclones where community diversity decreased, most of the samples within these eddies also had a decrease in functional diversity (Fig. S7). The anticyclone in the western gyre at 18°S concurrently experienced a decrease in nutrient supply and phosphate concentrations, while the anticyclone in the Bay of Bengal at 8°N concurrently experienced an increase in nutrient supply but no measurable changes in nutrient concentrations (Figs. S1 and S2). When combined with the community diversity analyses, these analyses suggest that when cyclones have specific characteristics and sufficiently alter nutrient supply/nutrient concentrations, this may lead to increases in community diversity and functional diversity.

### Ecological mechanisms of eddy-driven changes to bacterial community diversity

The ecological mechanism shaping community diversity was eddy specific. Because of their known environmental partitioning, *Prochlorococcus* ecotypes were examined in surface waters along the eastern transect to determine if vertical dispersal had occurred within cyclones. The high-light I (HLI) clade, which is adapted to high light and low-temperature conditions, was dominant in a portion of the eastern gyre, but the high-light II (HLII) clade, which is adapted to high light and high temperatures, dominated throughout the rest of the eastern Indian Ocean (Fig. S8). The absence of low-light *Prochlorococcus* clades, which are usually in higher abundances within deeper waters, indicated that significant vertical dispersal to the surface ocean was unlikely within these cyclonic eddies. To distinguish the impact of environmental selection versus lateral dispersal within specific eddies, partial Mantel tests were performed on two large cyclones and two large anticyclones (Table 2). The first cyclone originated off an island’s coast in the eastern Bay of Bengal and traveled westward to the center of the Bay of Bengal at 10°N (Fig. S9). Within this cyclone, community structure significantly correlated with geographic distance between samples (Mantel *r* = 0.19) but not with environmental variation, suggesting that there may have been a small dispersal effect. The second cyclone originated in the center of the Bay of Bengal where it remained at 6°N (Fig. S9). Within this cyclone, community structure significantly correlated with environmental variation (Mantel *r* = 0.54) but not with geographic distance, indicating that environmental selection may have occurred. The first anticyclone originated in the eastern equatorial region where it remained at 4°N (Fig. S9). Within this anticyclone, community structure significantly correlated with geographic distance (Mantel *r* = 0.59) but not with environmental variation. The second anticyclone originated in the Arabian Sea where it remained at 15°N (Fig. S9). Within this anticyclone, there was no significant relationship of community structure with environmental variation or with geographic distance between samples. Overall, it was observed that the biome is the primary determinant of community diversity with lateral dispersal and environmental selection potentially playing a secondary role within specific cyclones, but the influence of these ecological mechanisms in anticyclones remains unclear.

**Table 2:** Partial Mantel tests of community diversity with geographic distance and environmental variation within 4 large eddies

Transect	Polarity	Eddy ID	Source of origin	Latitude	Geographic distance		Environmental variation	
					Mantel *r*	*P*-value	Mantel *r*	*P*-value
Eastern	Cyclone	Cyc9 (S–N)	Island/coastal	10°N	0.19	.030*	−0.06	.700
Eastern	Cyclone	Cyc8	Open ocean	6°N	0.01	.460	0.54	.002*
Eastern	Anticyclone	Anticyc4	Open ocean	4°N	0.59	<.0001*	−0.16	.830
Western	Anticyclone	Anticyc12	Open ocean	15°N	0.06	.330	0.23	.090

* indicates statistical significance.

### Impact of eddies on bacterial community composition

**Figure 4:**
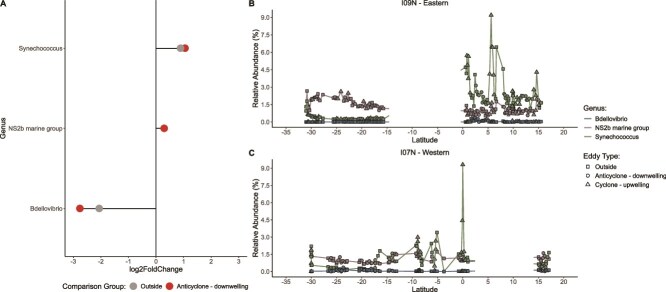
Changes in taxa within eddies. (**A**) Log_2_ fold change of differentially abundant genera. Latitudinal trends in differentially abundant genera along the eastern (**B**) and western (**C**) Indian Ocean.

Taxonomic composition primarily varied by biome with differential abundance of specific genera associated with eddy type. The total relative abundance of dominant taxa significantly varied by biome (*P* < .05). Across all biomes, *Prochlorococcus*, SAR11 Clade 1a, SAR11 Clade 1b, and *Candidatus* Actinomarina were the most abundant genera, and combined, they dominated community composition. Noticeable differences in taxonomic composition across the biomes included the presence of *Thiomicrorhabdus* in the western gyre, the presence of *Ascidiaceihabitans* in the western and eastern gyres, higher abundances of *Formosa* in the eastern gyre, higher abundances of *Alteromonas* in the western equatorial region, higher abundances of *Pirellula* in the eastern equatorial region, and higher abundances of *Vibrio* across the eastern Indian Ocean compared to the western Indian Ocean with the highest abundances of *Vibrio* observed in the Bay of Bengal. The total relative abundance of dominant taxa did not significantly vary by eddy type (*P* > .05); however, large shifts in community composition of individual taxa were observed within certain eddies. For example, a cyclone in the western equatorial region at 9°S had increased abundance of *Alteromonas* but decreased abundances of SAR11 Clade 1a and *Prochlorococcus*, while the latitudinal transect of a cyclone in the Bay of Bengal at 10°N had a large increase in *Vibrio* and decrease in *Prochlorococcus* (Fig. S10). Furthermore, within cyclones, two genera (*Synechococcus* and NS2b marine group) were found in significantly higher abundances (adj. *P* < .05) and one genus (*Bdellovibrio*) was found in significantly lower abundances (adj. *P* < .05) compared to anticyclones and non-eddy samples (Fig. 4). Increases in *Synechococcus* were commonly seen within cyclones in the Bay of Bengal, while the NS2b marine group was more abundant within cyclones in the southern gyre (Fig. 4). Thus, variations in the taxonomic composition were primarily influenced by the biome with notable shifts observed within individual eddies.

### Impact of eddies on bacterial functional categories and composition

There were minor variations in COG functional categories across the Indian Ocean. The proportion of unigenes assigned to each COG category significantly varied by biome (*P* < .05) but not by eddy type (*P* > .05) (Fig. S11). However, there were minor, significant differences in the proportions of unigenes assigned to specific COG functional categories between eddy types along the western and eastern transects (Table S2). For example, along the western transect, COG category D (cell cycle control, cell division, chromosome partitioning) was significantly different between cyclones and anticyclones (adj. *P* = .012), and along the eastern transect, COG category H (coenzyme transport and metabolism) was significantly different between cyclones and non-eddy samples (adj. *P* = .040). Therefore, eddies had a nuanced impact on broad categories of function.

**Figure 5:**
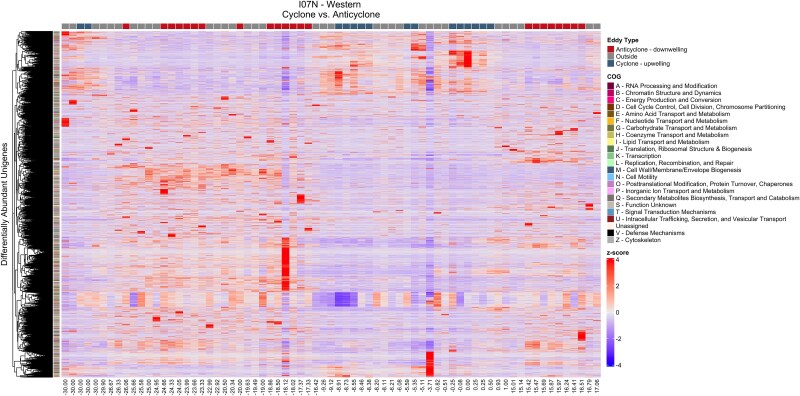
Heatmap of differentially abundant unigenes found in a pairwise comparison of eddy types in the western Indian Ocean. Each row represents a unigene, and each column represents a sample labeled by its latitude. Unigenes are color scaled according to their *z*-scores to highlight relative increases or decreases. The dendrogram clusters unigenes based on their normalized, scaled abundances to aid in identifying patterns across samples. Colored boxes above the heatmap indicate the eddy type associated with each sample, while colored boxes on the side denote the COG category assigned to each unigene.

Each eddy had a unique composition of differentially abundant unigenes (Fig. 5 and Figs. S12–S16). The top three COG functional categories that the differentially abundant unigenes were assigned to were S (function unknown), M (cell wall, membrane, envelope biogenesis), and E (amino acid transport and metabolism). The differentially abundant unigenes had a variety of functional annotations that were metabolically or biologically important such as Fe-transport, N_2_-fixation, hexosamine metabolism, photosystems, osmoprotectants, quorum sensing, CRISPR, and phage integrases (Table S1). Overall, eddies exhibited distinct profiles of differentially abundant unigenes that have metabolic and biological significance.

Eddies had different suites of metabolically and biologically relevant unigenes that corresponded to biome and eddy parameters (Table S3; PCA plots not shown). For example, for Fe-transport unigenes, P-stress had the strongest correlation (*P* < .001, *r* = 0.775) with PC1, and eddy age had the strongest correlation (*P* < .001, *r* = 0.467) with PC2. For N_2_-fixation unigenes, temperature had the strongest correlation (*P* < .001, *r* = −0.368) with PC1, while eddy age had the strongest correlation (*P* < .001, *r* = −0.415) with PC2. Overall, these analyses showed that typically the strongest correlations occurred with biome parameters, while eddy parameters had weaker correlations. This provided further support that the biome is the primary determinant of bacterial function with eddies playing an important secondary role.

## Discussion

We hypothesized that eddy-induced disturbance would have a substantial impact on bacterial diversity. However, we found minimal support for this hypothesis. Instead, we observed that eddies mostly played a weak, secondary role in shaping diversity. This result not only challenges our initial hypothesis but also contradicts the established paradigm, which views eddies as significant disturbances. Other studies have shown significant decreases of community diversity within singular cyclones [15, 42], demonstrating that some eddies can cause strong disturbances. Here, inconsistent changes in diversity throughout eddies were observed. Development stage and intensity of cyclones have been hypothesized to be important in contributing to varying biological responses within cyclones [43]. We observed that while age, amplitude, and speed were important predictors of community diversity, the relationship of these predictors with diversity was weak, and a consistent signal of change aligning with these eddy characteristics was rare. Overall, our study showed that the average impact of an eddy disturbance was limited compared to biome dynamics in shaping bacterial communities.

Second, we hypothesized that eddies would impact bacterial communities through the combined processes of environmental selection and dispersal. This hypothesis was not supported by the analyses that could be performed. There was also no evidence of substantial vertical dispersal in any of the eastern Indian Ocean cyclones, which contradicts previous studies [13–15]. There was some evidence of potential lateral dispersal within one cyclone in the Bay of Bengal at 10°N, where changes in community structure weakly correlated with changes in geographic distance between samples. The degree of lateral dispersal may have been influenced by the point of eddy origin, which aligns with prior observations where lateral entrainment of communities from different points of origin into offshore waters impacted community composition [12, 44] and function [17]. Additionally, there was some evidence of environmental selection within a cyclone in the Bay of Bengal at 6°N where changes in community structure moderately correlated with changes in environmental conditions. Within this cyclone, there were decreases in phosphate and silicate concentrations that coincided with increases in *Synechococcus* and with decreases in community and functional diversity. A similar response in *Synechococcus* was observed within a cyclone in the South China Sea during the intensifying stage, and it was hypothesized that the increased abundance was due to the upward flux of inorganic nutrients caused by the formation of the eddy [43]. These observations align with the first ecological framework outlined in the Introduction in which physical displacement of water masses triggers changes in the geochemical environment, subsequently selecting for specific phytoplankton communities which alters the bacterial community. However, evidence for the influence of lateral dispersal and/or environmental selection was scarce, and it remains inconclusive how these mechanisms operate independently or interactively to shape bacterial communities within eddies.

Lastly, we investigated how taxonomy and function changed across biomes and eddy types. *Prochlorococcus* and the SAR11 clades are ubiquitous across the Indian Ocean and dominate bacterial community composition, while *Synechococcus* is typically found in low abundances [21]. Within one anticyclone in the western gyre at 18°S, we observed that *Synechococcus* abundances decreased while *Prochlorococcus* increased. This same pattern was also observed within an anticyclone in the South China Sea [16]. Here, several cyclones displayed the opposite pattern in picophytoplankton abundance. *Synechococcus* increased substantially within Bay of Bengal cyclones, while *Prochlorococcus* decreased in cyclones throughout the basin. Similarly, within a cyclone south of the Canary Islands, *Synechococcus* biomass was higher in the surface ocean compared to *Prochlorococcus* [45]. However, these picophytoplankton dynamics may be dependent on the specific conditions within an eddy. For example, *Synechococcus* increased in a cyclone in the intensifying stage, while a cyclone in the decaying stage was dominated by *Prochlorococcus* [43]. Combined, these observations align with the idea that eddies can either favor k-strategist or r-strategist cell types under specific conditions [11]. Anticyclones can enhance microbial functions such as N_2_-fixation [46] and sulfur-cycling [17]. Here, each eddy had its own unique composition of important functions that were found in varied abundances and that had stronger associations with biome properties than eddy characteristics. Our observations along with prior studies demonstrate the variability in taxonomic and functional responses within and between eddy types and emphasize the importance of understanding eddy stage of formation and biome attributes in interpreting bacterial responses.

Within this study, a Eulerian framework was implemented to define eddy boundaries and perform sampling, which has several important implications. Defining eddy boundaries using sea level anomaly (SLA) assumes eddy coherency (i.e. trapping and transport of fluid) [47]. However, eddy boundaries may be actively mixing with the surrounding waters. A comparative analysis of rotationally coherent Lagrangian vortices (RCLV) with SLA-identified eddies in the North Pacific Subtropical Gyre showed that approximately half of SLA-identified eddies are not coherent [47]. Thus, many SLA-identified eddies are actively mixing with nearby waters, possibly “diluting” the microbial signal. Additionally, RCLV eddies are on average 4.7 times smaller than SLA-identified eddies. This size difference alters which samples are being defined as inside versus outside of an eddy and thus alters the observed microbial outcomes. Second, the Eulerian framework used targeted *in situ* sampling to collect bacterial communities at single locations at a single time point. The benefit of this approach is that a wide range of variation can be captured within a single study. Using the Eulerian framework, we were able to study 26 individual eddies distributed across six different biomes. However, the number of eddies that intersected with the GO-SHIP transects varied by biome, creating an imbalance that may influence the interpretation of biome versus eddy effects on bacterial communities, particularly in biomes with few eddies. An additional limitation of the Eulerian approach is that it was difficult to identify the ecological mechanisms driving changes in the communities. Employing a Lagrangian approach, where samples are collected laterally and vertically from the point of eddy origin through its development stages, would allow the dominant ecological mechanism to be identified for that specific eddy. These differences emphasize the importance of further methodological refinement to improve our understanding of microbial responses to mesoscale eddy disturbances.

Climate change is leading to the expansion and poleward shift of oligotrophic regions as well as the intensification and geographic shift of eddy fields. The ongoing expansion of oligotrophic regions has been linked to increasing sea surface temperatures [48–50]. Additionally, oligotrophic gyres are predicted to shift poleward in a warming climate [51]. Concurrently, CMIP6 simulations predict that eddy kinetic energy will intensify and have a poleward shift within many eddy-rich regions [52]. Thus, we may observe a geographic overlap in the expansion of oligotrophic regions with intensifying eddy fields. How phytoplankton and bacterial communities will respond to this convergence of conditions remains inconclusive. There is some evidence that phytoplankton biomass [53] and net primary productivity [54] will decline. However, phytoplankton communities are complex and have adaptive capabilities that may buffer the impacts of changing temperature and nutrient conditions [55]. The Indian Ocean is largely oligotrophic with numerous eddy fields and thus can inform on how bacterial communities will respond to oligotrophication and eddy intensification in other ocean basins. Results presented here indicate that changes to the biome (i.e. oligotrophication) will be the largest contributing factor to changes in bacterial diversity with small, variable impacts of eddy presence. Thus, this study reveals valuable insights into how bacterial communities may respond to climate change.

## Supplementary Material

MLB_IndianOceanEddies_ISME_Supplemental_Figures_ycaf110

MLB_IndianOceanEddies_ISME_042924_Supplemental_Tables_ycaf110

MLB_IndianOceanEddies_ISME_Supplemental_Methods_wRef_ycaf110

## Data Availability

The data analyzed in the present study are accessible in the following open repositories. GO-SHIP cruise metadata are available via cchdo.ucsd.edu. Raw sequence reads are available via NCBI SRA at BioProject ID: PRJNA656268 (16S rRNA and short-read metagenomes) and at BioProject ID: PRJNA522445 (rpoC1).
