# Supplementary material for: Roles of physical disturbance and biome properties in shaping microbial communities within Indian Ocean eddies
Source: ISME Commun. 2025 Jul 2;5(1):ycaf110. doi: 10.1093/ismeco/ycaf110 (PMC12306440; doi:10.1093/ismeco/ycaf110)
Supplement: MLB_IndianOceanEddies_ISME_Supplemental_Figures_ycaf110 [file mlb_indianoceaneddies_isme_supplemental_figures_ycaf110.docx]

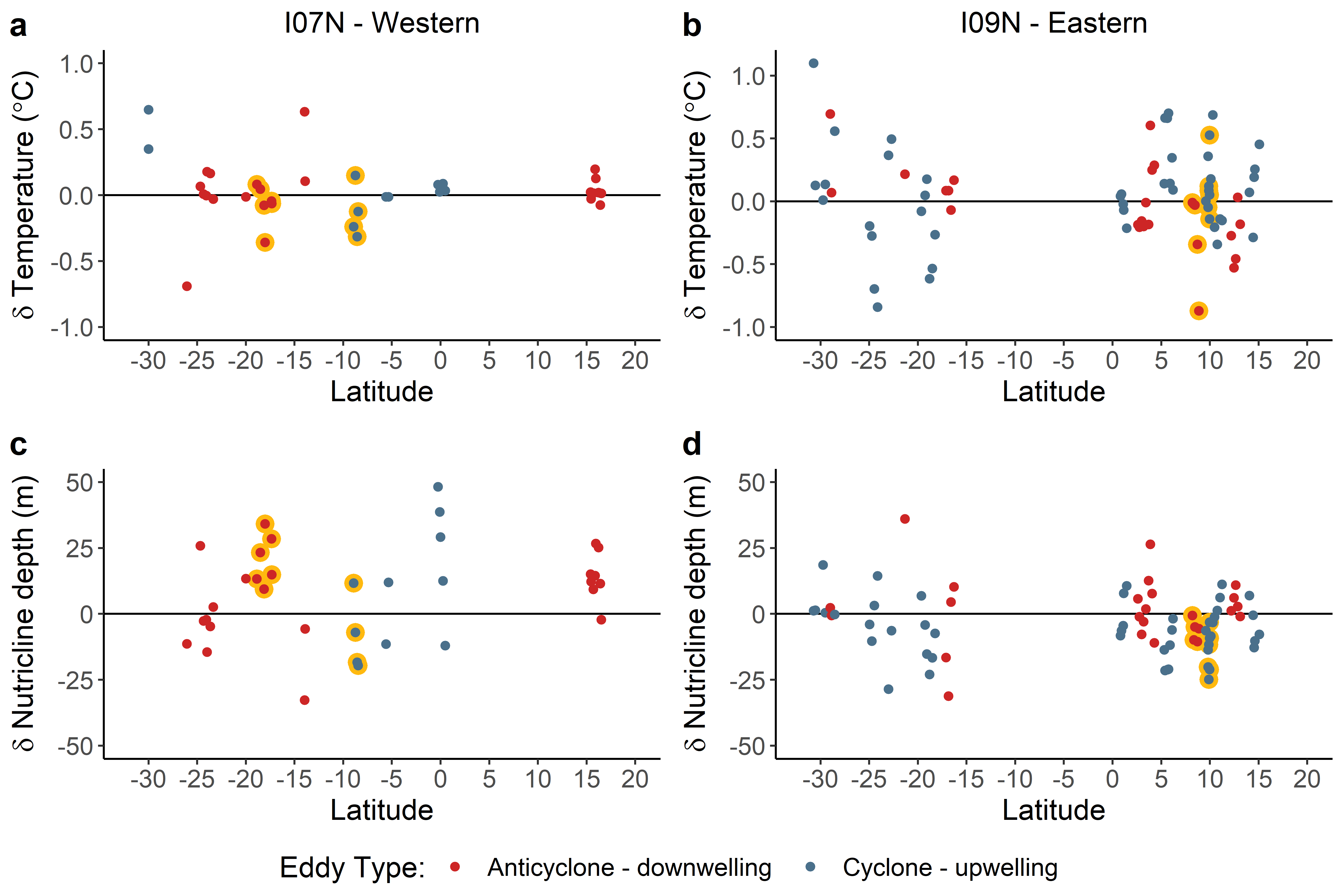


**Supplemental Figure 1: Environmental changes within eddies.** (**a** and **b**) Changes in temperature and (**c** and **d**) nutrient supply within each eddy as compared to the average values of the nearest control samples. Decreases in nutricline depth indicate a shallowing of the nutricline and thus an increase in nutrient supply, whereas increases in nutricline depth indicate a deepening of the nutricline and thus a decrease in nutrient supply. Points highlighted in yellow indicate samples within eddies that are discussed in detail within the text.


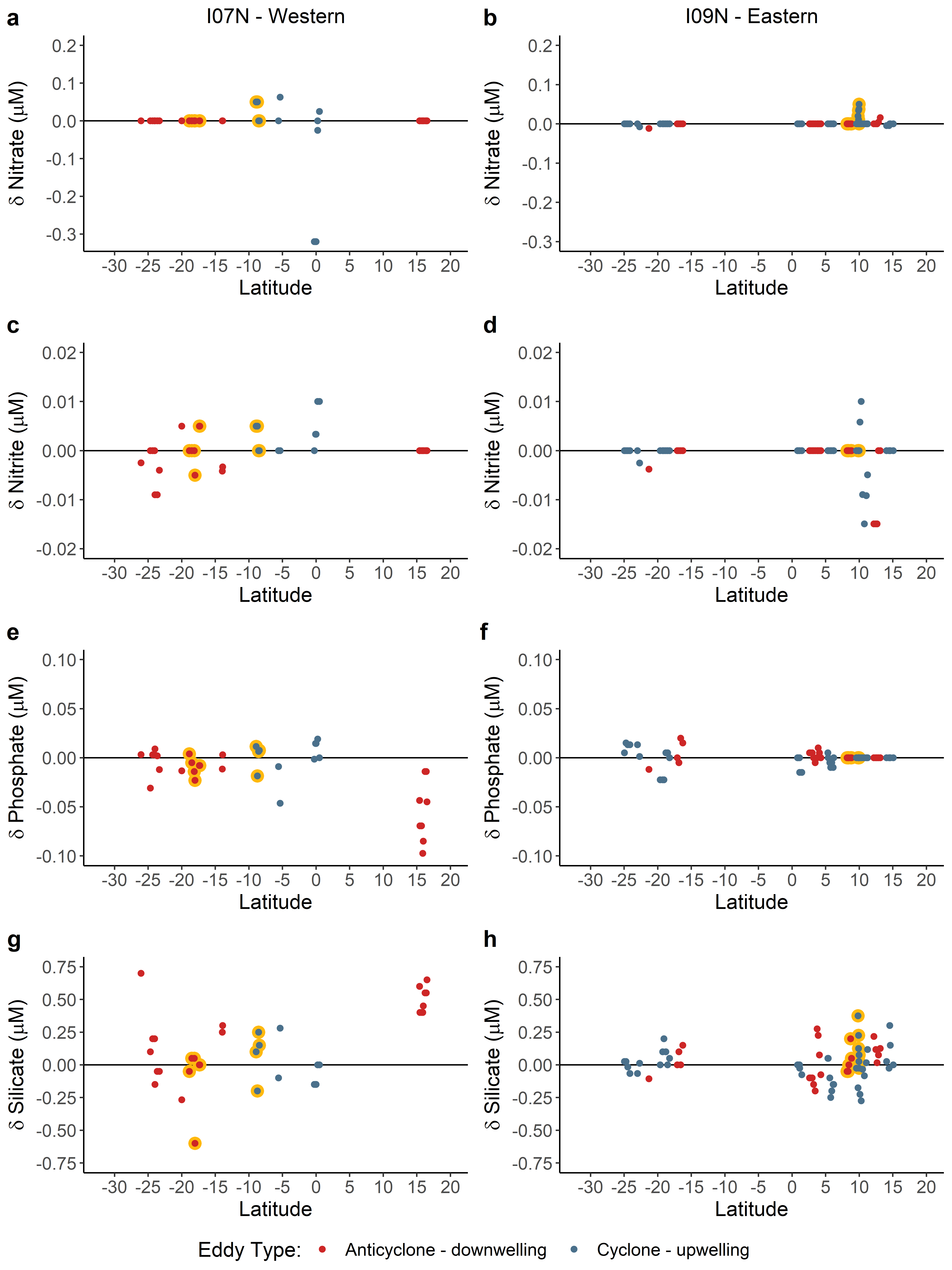


**Supplemental Figure 2: Geochemical changes within eddies.** (**a** and **b**) Changes in nitrate (**c** and **d**) nitrite (**e** and **f**) phosphate and (**g** and **h**) silicate concentrations (µM) within each eddy as compared to the average values of the nearest neighbor control samples. Points highlighted in yellow indicate samples within eddies that are discussed in detail within the text.

*
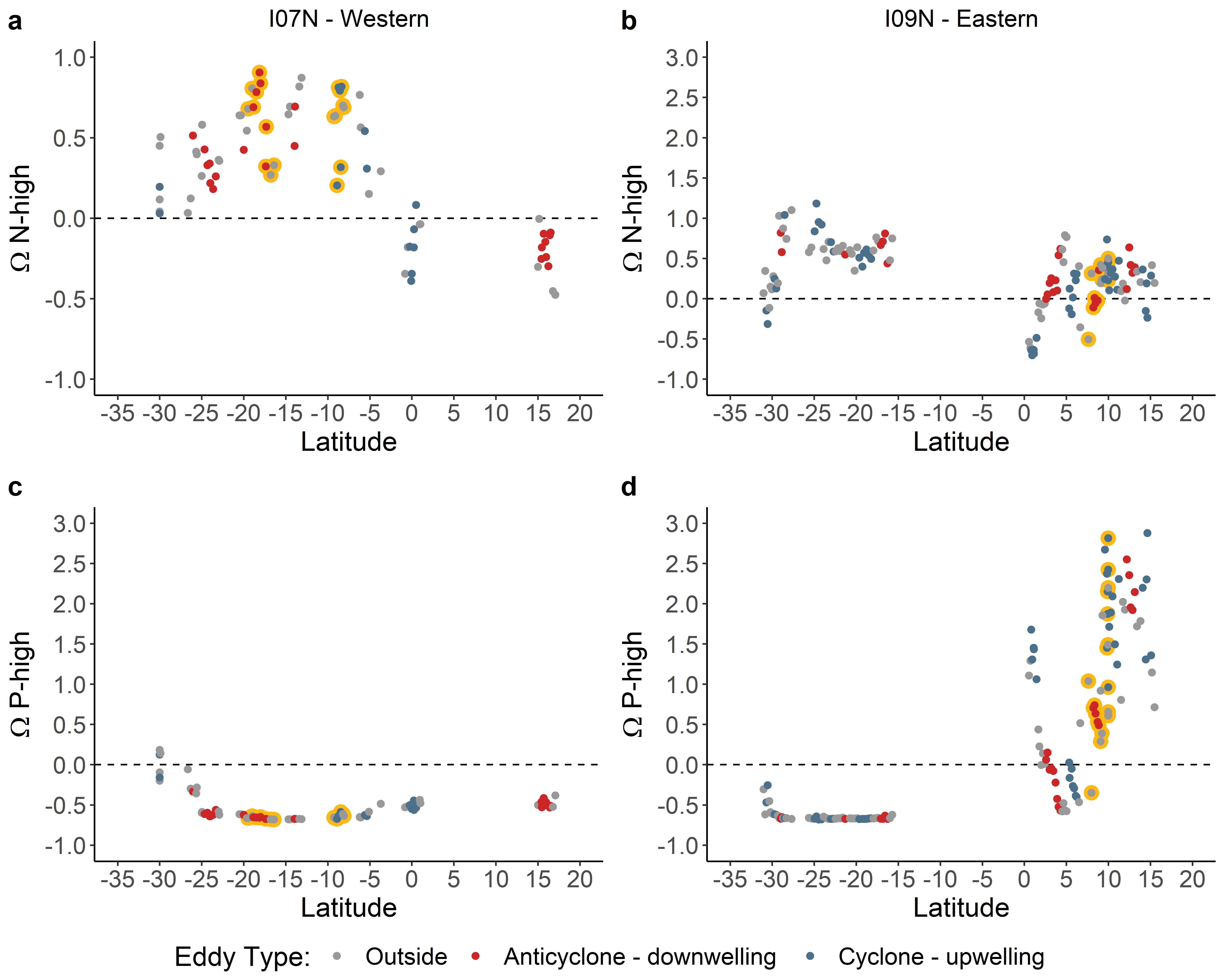
*

**Supplemental Figure 3: Nutrient stress within eddies.** *Prochlorococcus* gene content indicates N-stress throughout the basin, except in the northeastern Indian Ocean where there is a shift to P-stress. (**a** and **b**) Nutrient stress index of high N-stress and (**c** and **d**) high P-stress. Points highlighted in yellow indicate samples within eddies that are discussed in detail within the text.


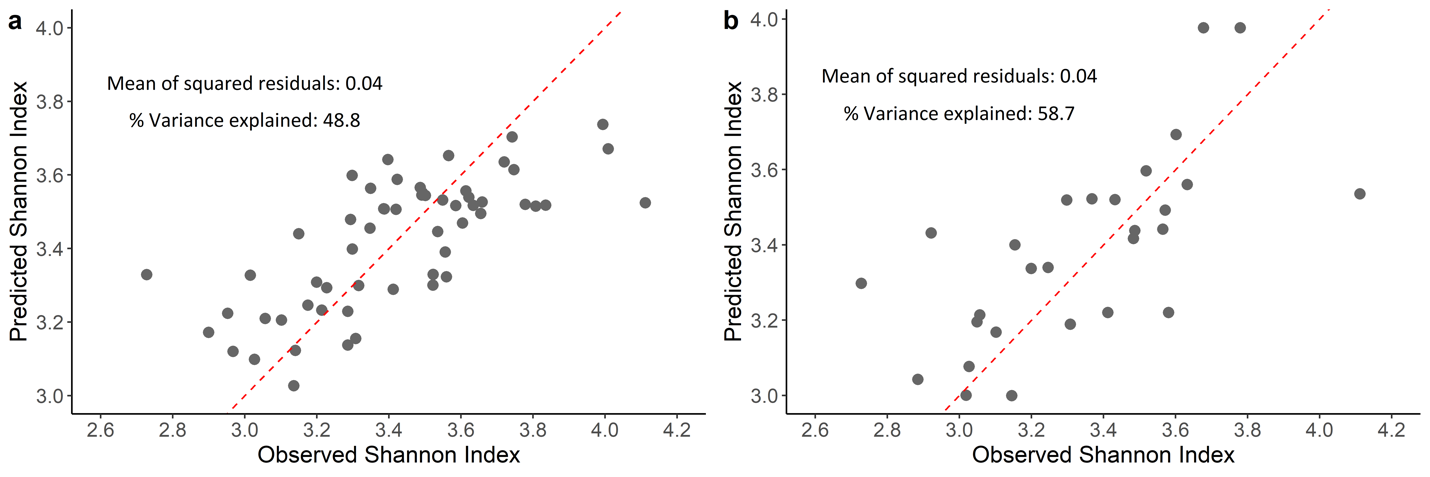


**Supplemental Figure 4: Model evaluation of random forest regressions.** Predicted community diversity calculated by the random forest regression model versus observed community diversity for (**a**) all samples and (**b**) only eddy samples.


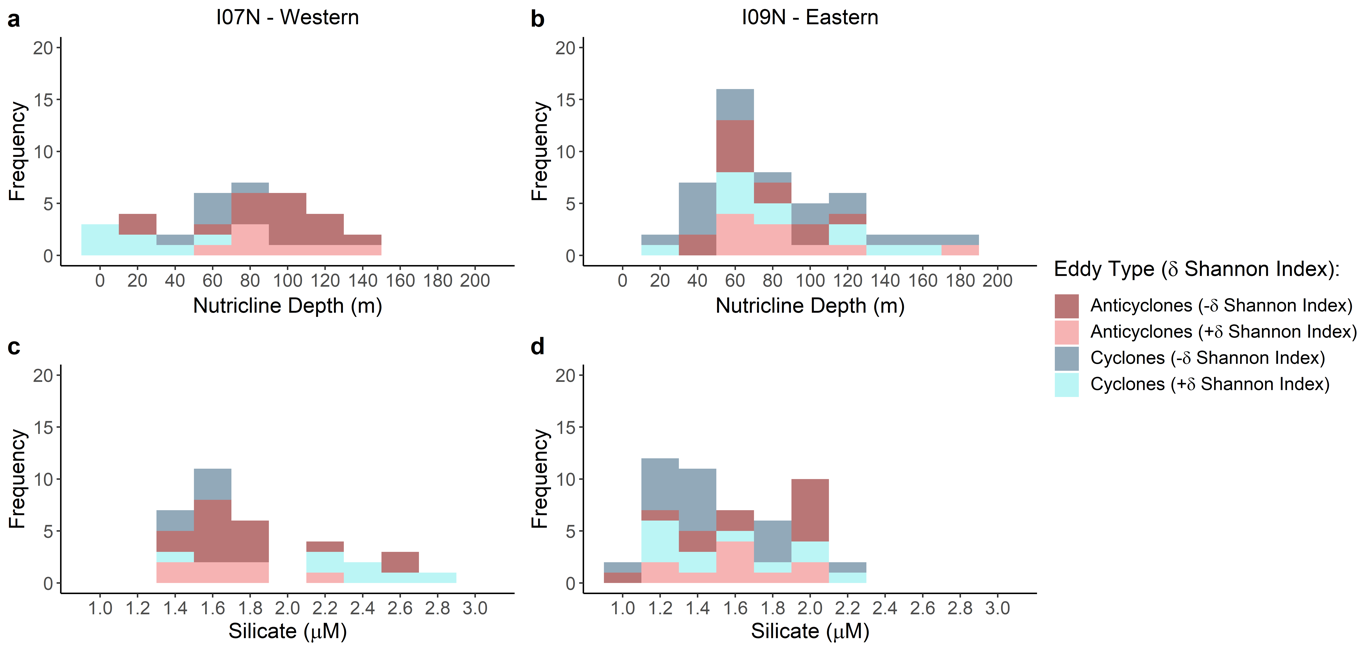


**Supplemental Figure 5: Correspondence of changes in community diversity within eddies with environmental conditions.** (**a** and **b**) Nutricline depth (m) and (**c** and **d**) silicate concentrations (µM) correspond with changes in community diversity within cyclonic eddies along the western transect.


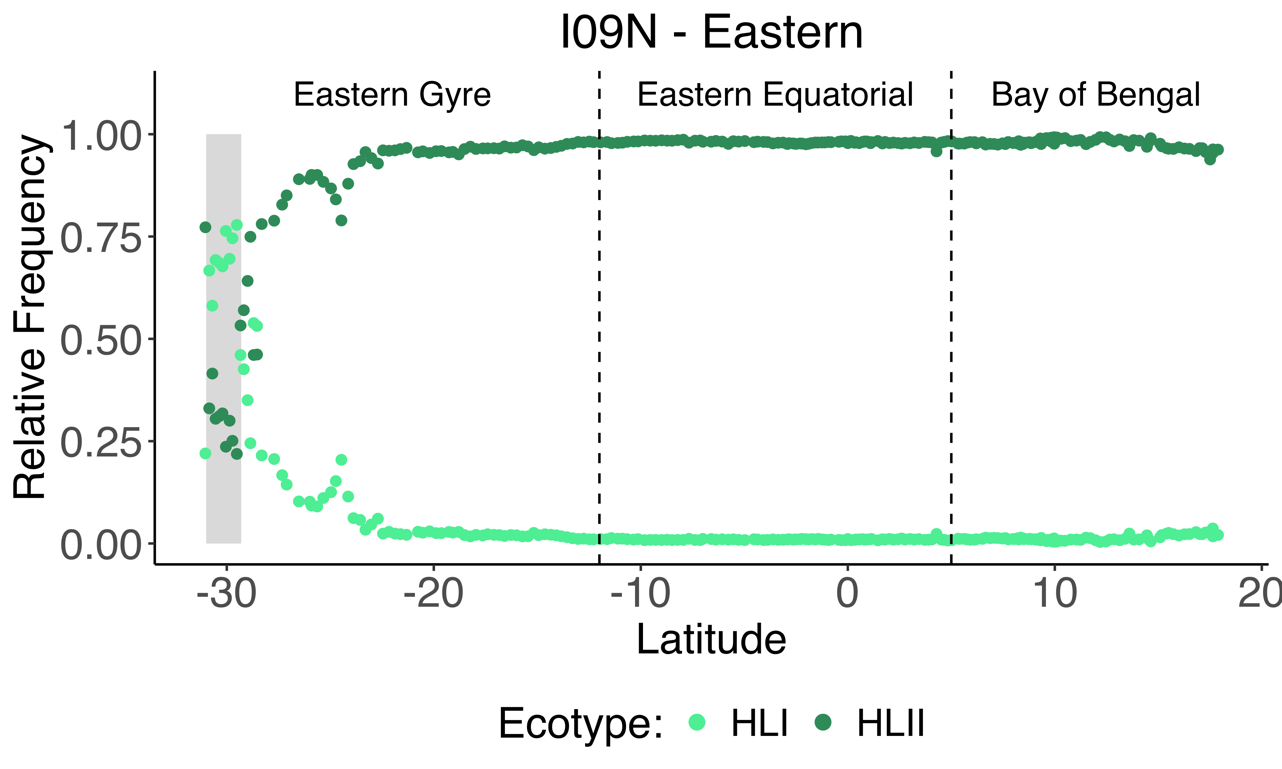


**Supplemental Figure 6: Relative frequencies of *Prochlorococcus* ecotypes across the eastern Indian Ocean**. High-light I (HLI) and high-light II (HLII) ecotypes were the dominant ecotypes with the shaded gray box indicating the specific location where HLI was dominant in the southeastern gyre. A relative frequency value of 1 is equivalent to 100% relative abundance. The absence of low-light ecotypes indicates a lack of significant vertical dispersal to the surface ocean.


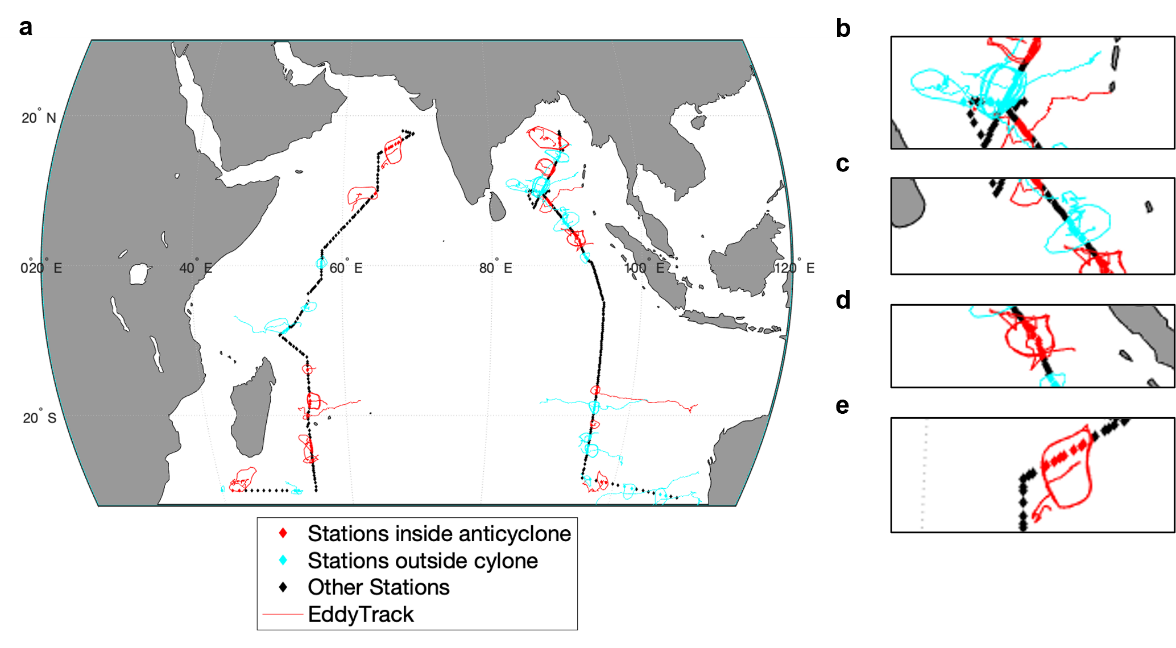


**Supplemental Figure 7: Origin and travel trajectories of eddies across the Indian Ocean.** (**a**) Origin and travel trajectories of all eddies. Origin and travel trajectory of (**b**) cyclone at 10°N in the Bay of Bengal (**c**) cyclone at 6°N in the Bay of Bengal (**d**) anticyclone at 4°N in the eastern equatorial and (**e**) anticyclone at 15°N in the Arabian Sea.


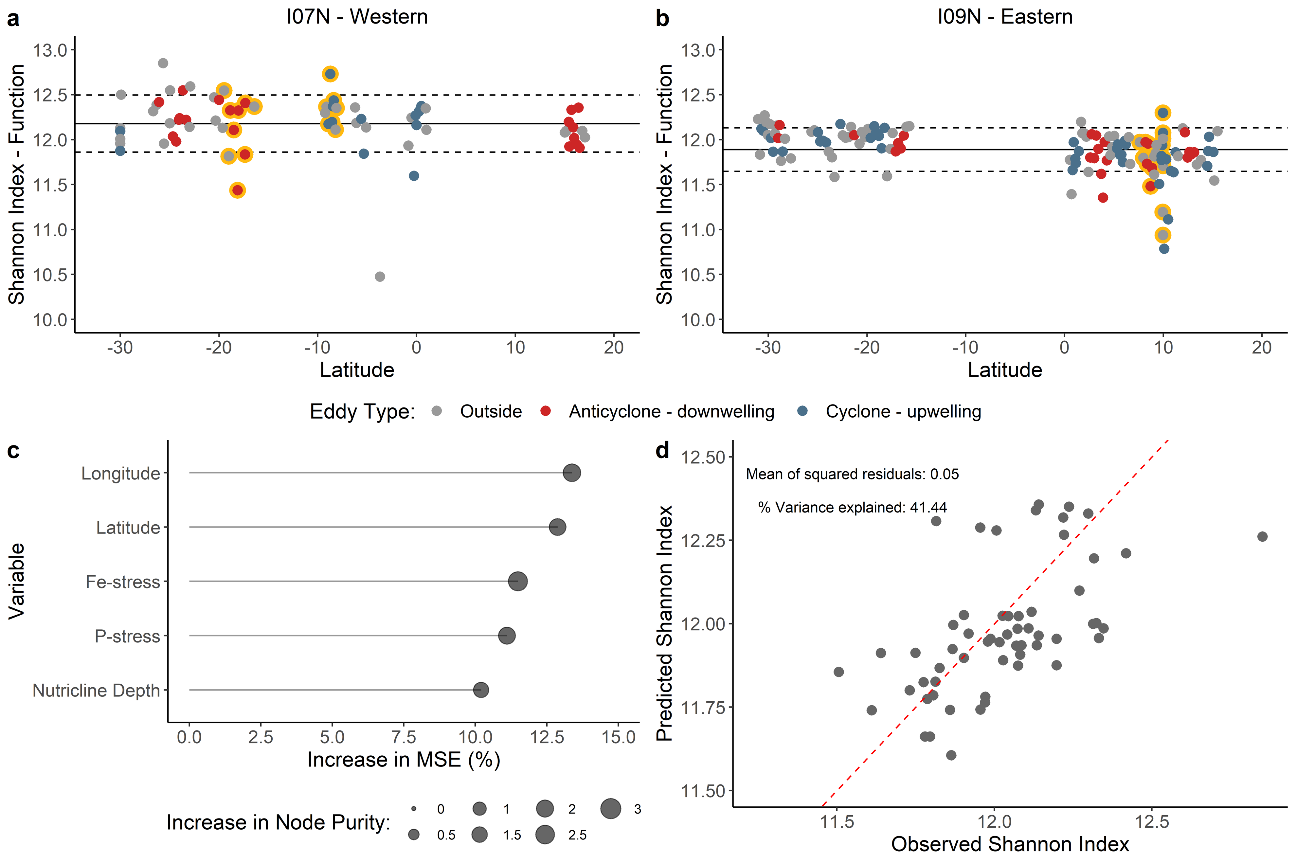


**Supplemental Figure 8: Spatial trends and best predictors of functional diversity.** (**a** and **b**) Longitudinal and latitudinal trends in functional diversity calculated from unigenes using the Shannon Index. Points highlighted in yellow indicate samples within eddies that are discussed in detail within the text. (**c**) Best predictors of functional diversity as determined through random forest regression. (**d**) Predicted functional diversity calculated by the random forest regression model versus observed functional diversity.


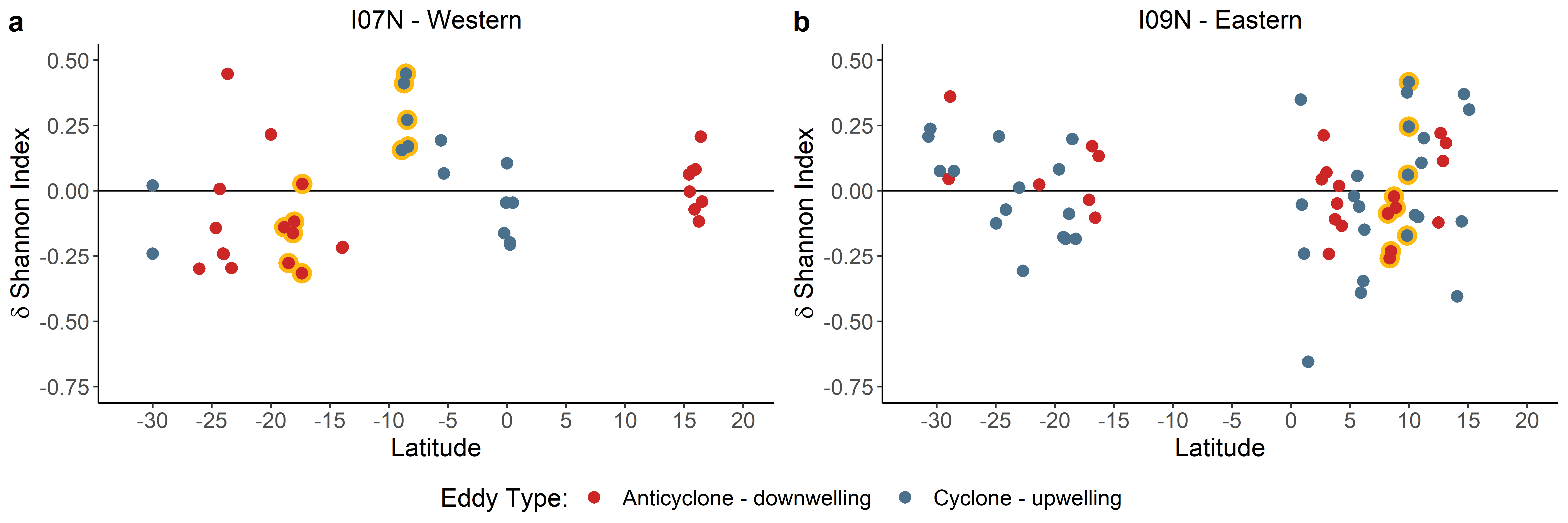


**Supplemental Figure 9: Changes in functional diversity within eddies**. Changes in functional diversity of unigenes within each eddy on the (**a**) western and (**b**) eastern transects as compared to the average values of the nearest neighbor control samples. Points highlighted in yellow indicate samples within eddies that are discussed in detail within the text.


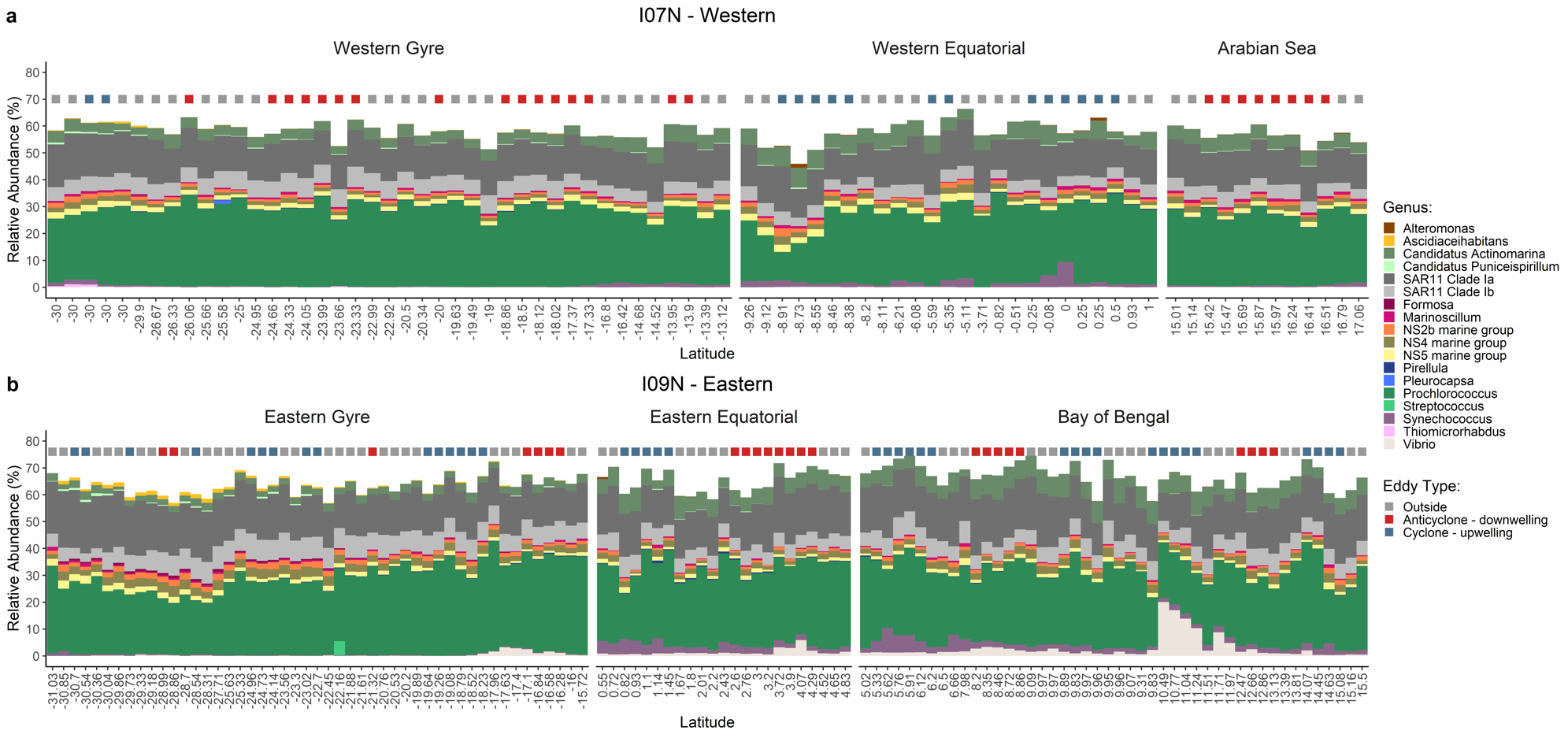


**Supplemental Figure 10: Community composition of dominant taxa across the Indian Ocean.** Relative abundance of dominant taxa (≥ 1% relative abundance) within eddy and non-eddy samples in the (**a**) western and (**b**) eastern Indian Ocean. Genera listed in the legend are ordered to match the top-to-bottom arrangement of colors in the stacked bar plots. Colored boxes above the stacked bars indicate sample type (i.e., cyclonic eddy, anticyclonic eddy, or non-eddy).


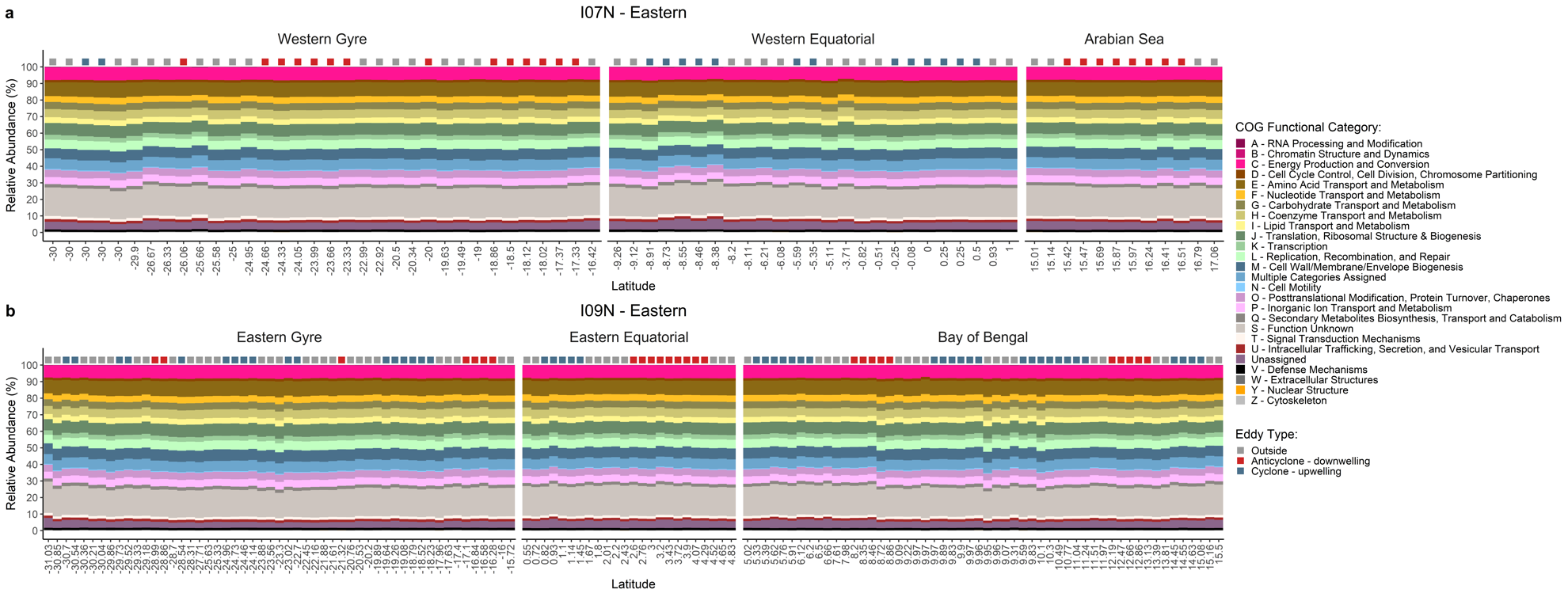


**Supplemental Figure 11: COG functional categories across the Indian Ocean.** Relative abundance of COG functional categories in the (**a**) western and (**b**) eastern Indian Ocean eithin eddy and non-eddy samples. COG functional categories listed in the legend are ordered to match the top-to-bottom arrangement of colors in the stacked bar plots. Colored boxes above the stacked bars indicate sample type (i.e., cyclonic eddy, anticyclonic eddy, or non-eddy).

**
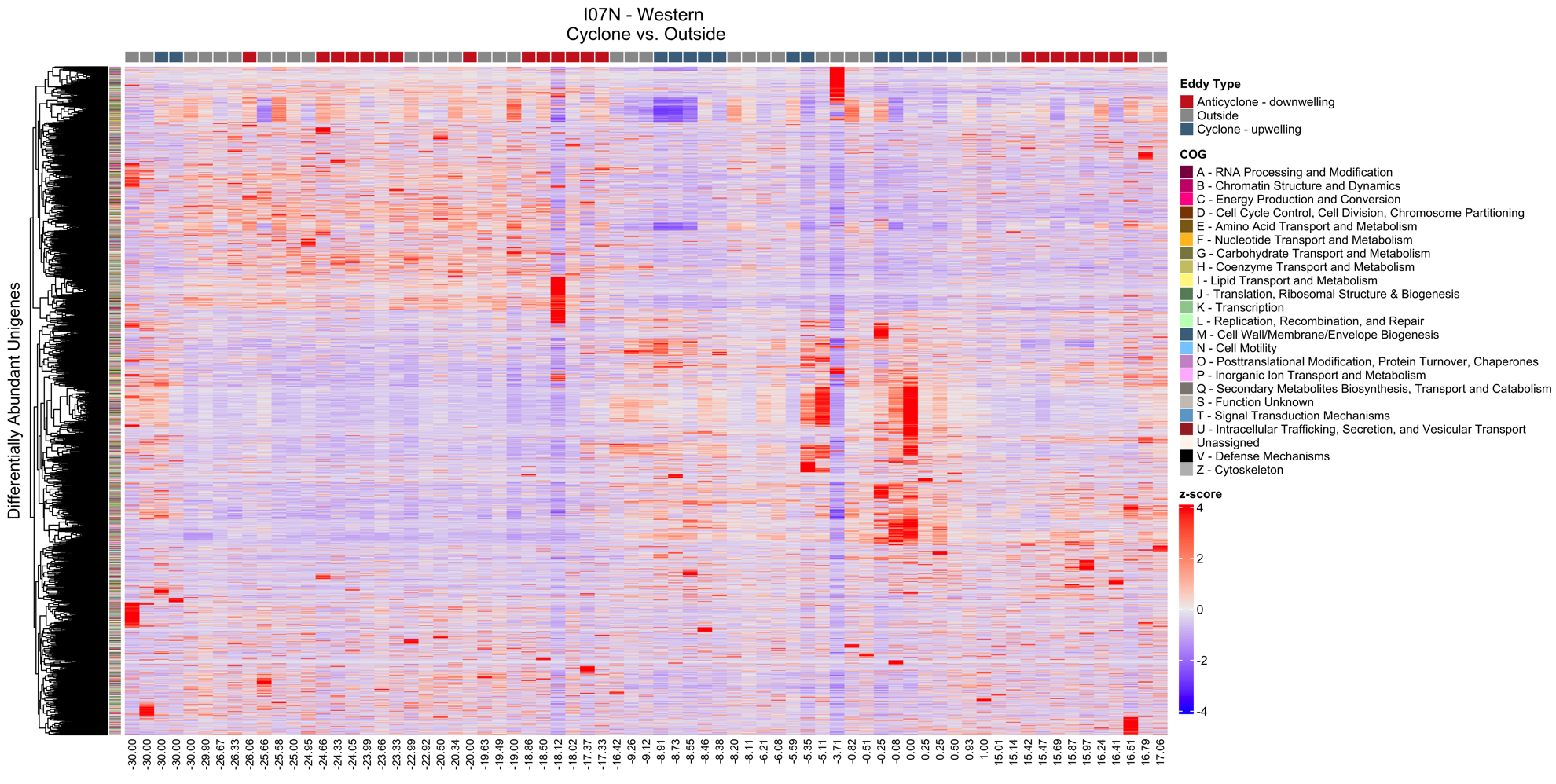
**

**Supplemental Figure 12: Heatmap of differentially abundant unigenes found in a pairwise comparison of cyclonic eddies and non-eddy samples in the western Indian Ocean.** Each row represents a unigene, and each column represents a sample labeled by its latitude. Unigenes are color-scaled according to their z-scores to highlight relative increases or decreases. The dendrogram clusters unigenes based on their normalized, scaled abundances to aid in identifying patterns across samples. Colored boxes above the heatmap indicate the eddy type associated with each sample, while colored boxes on the side denote the COG category assigned to each unigene.

**
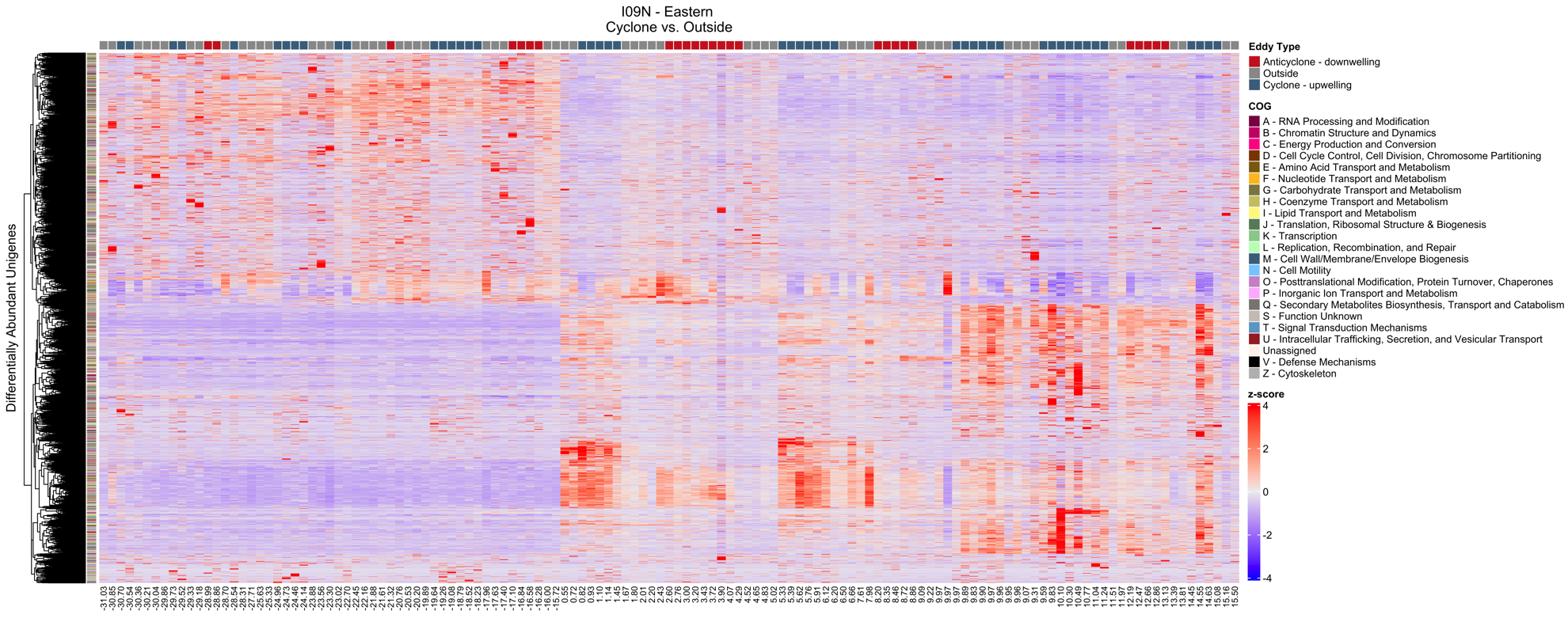
**

**Supplemental Figure 13: Heatmap of differentially abundant unigenes found in a pairwise comparison of cyclonic eddies and non-eddy samples in the eastern Indian Ocean.** Each row represents a unigene, and each column represents a sample labeled by its latitude. Unigenes are color-scaled according to their z-scores to highlight relative increases or decreases. The dendrogram clusters unigenes based on their normalized, scaled abundances to aid in identifying patterns across samples. Colored boxes above the heatmap indicate the eddy type associated with each sample, while colored boxes on the side denote the COG category assigned to each unigene.

**
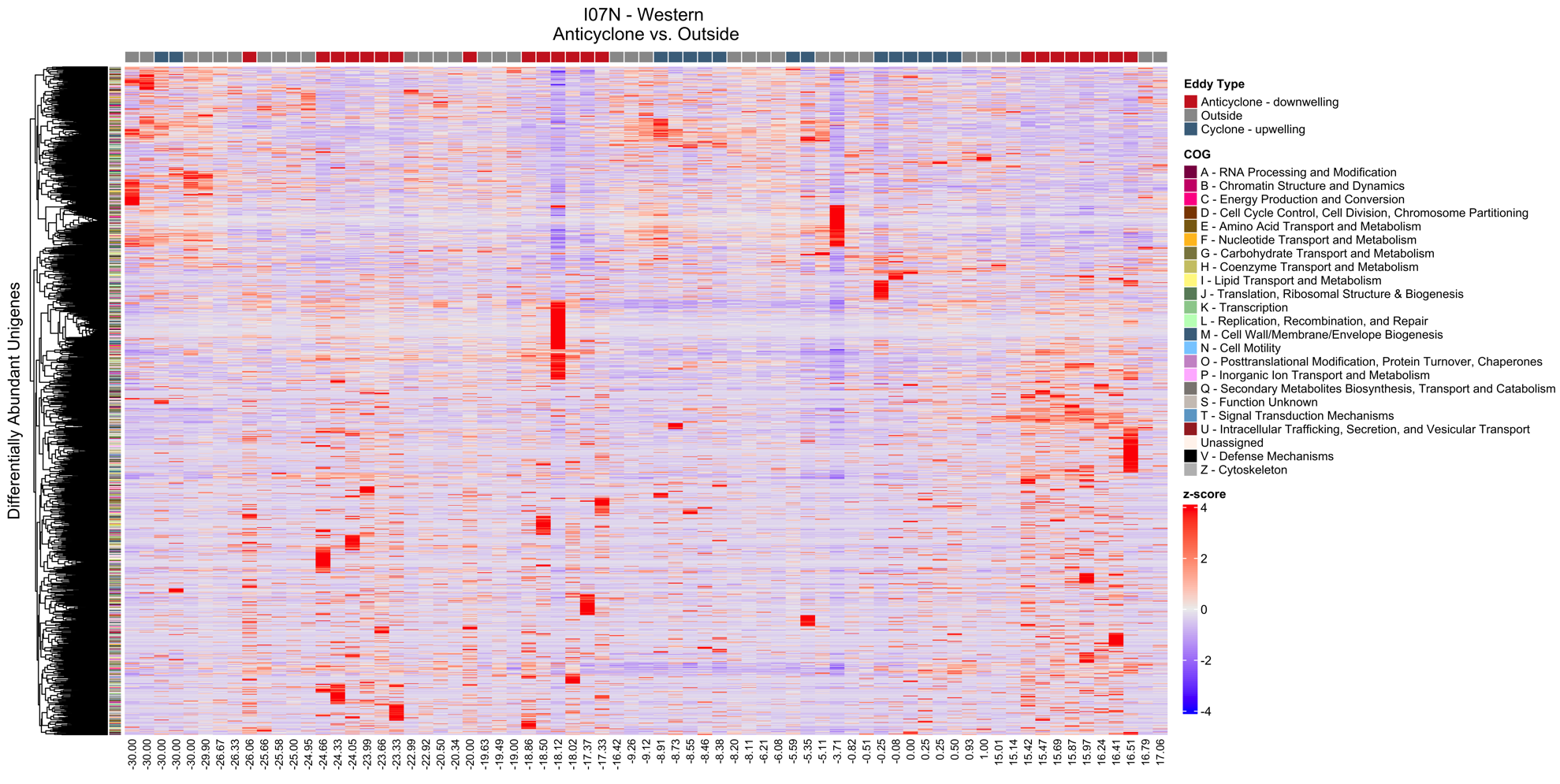
**

**Supplemental Figure 14: Heatmap of differentially abundant unigenes found in a pairwise comparison of anticyclonic eddies and non-eddy samples in the western Indian Ocean.** Each row represents a unigene, and each column represents a sample labeled by its latitude. Unigenes are color-scaled according to their z-scores to highlight relative increases or decreases. The dendrogram clusters unigenes based on their normalized, scaled abundances to aid in identifying patterns across samples. Colored boxes above the heatmap indicate the eddy type associated with each sample, while colored boxes on the side denote the COG category assigned to each unigene.

**
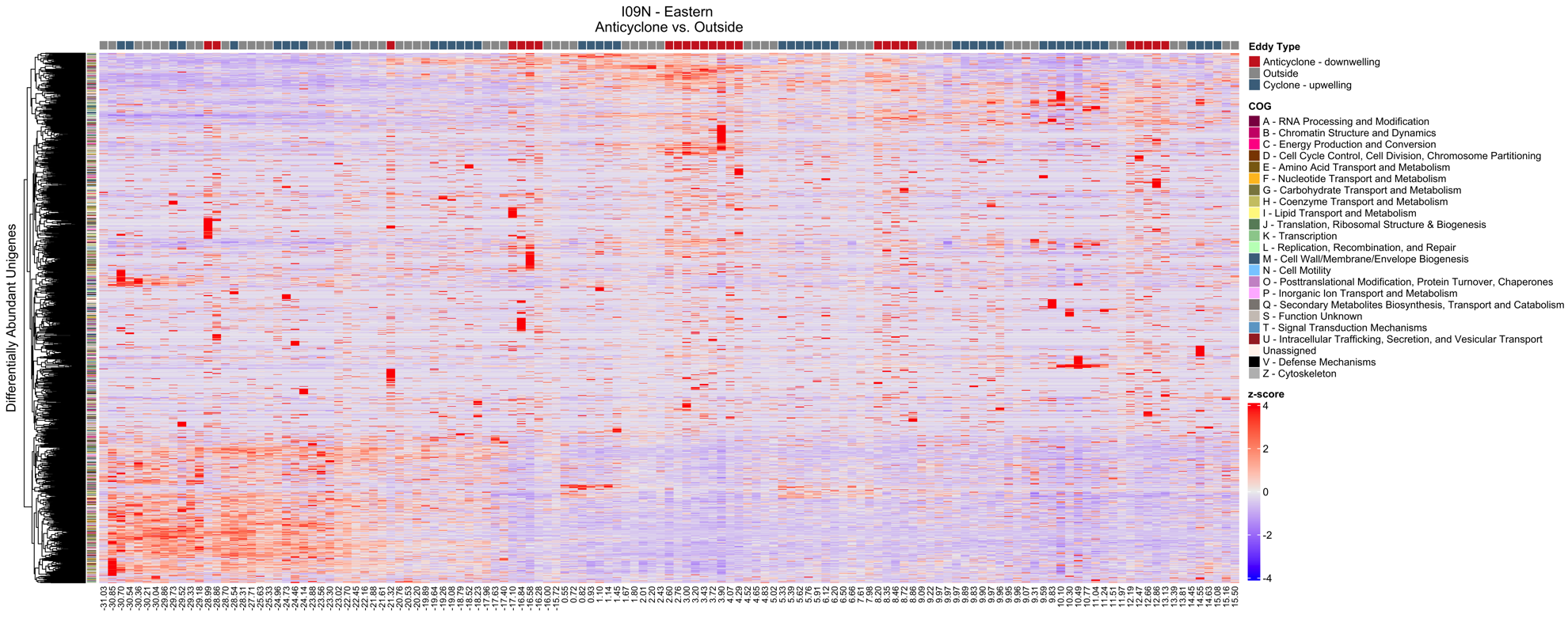
**

**Supplemental Figure 15: Heatmap of differentially abundant unigenes found in a pairwise comparison of anticyclonic eddies and non-eddy samples in the eastern Indian Ocean.** Each row represents a unigene, and each column represents a sample labeled by its latitude. Unigenes are color-scaled according to their z-scores to highlight relative increases or decreases. The dendrogram clusters unigenes based on their normalized, scaled abundances to aid in identifying patterns across samples. Colored boxes above the heatmap indicate the eddy type associated with each sample, while colored boxes on the side denote the COG category assigned to each unigene.

**
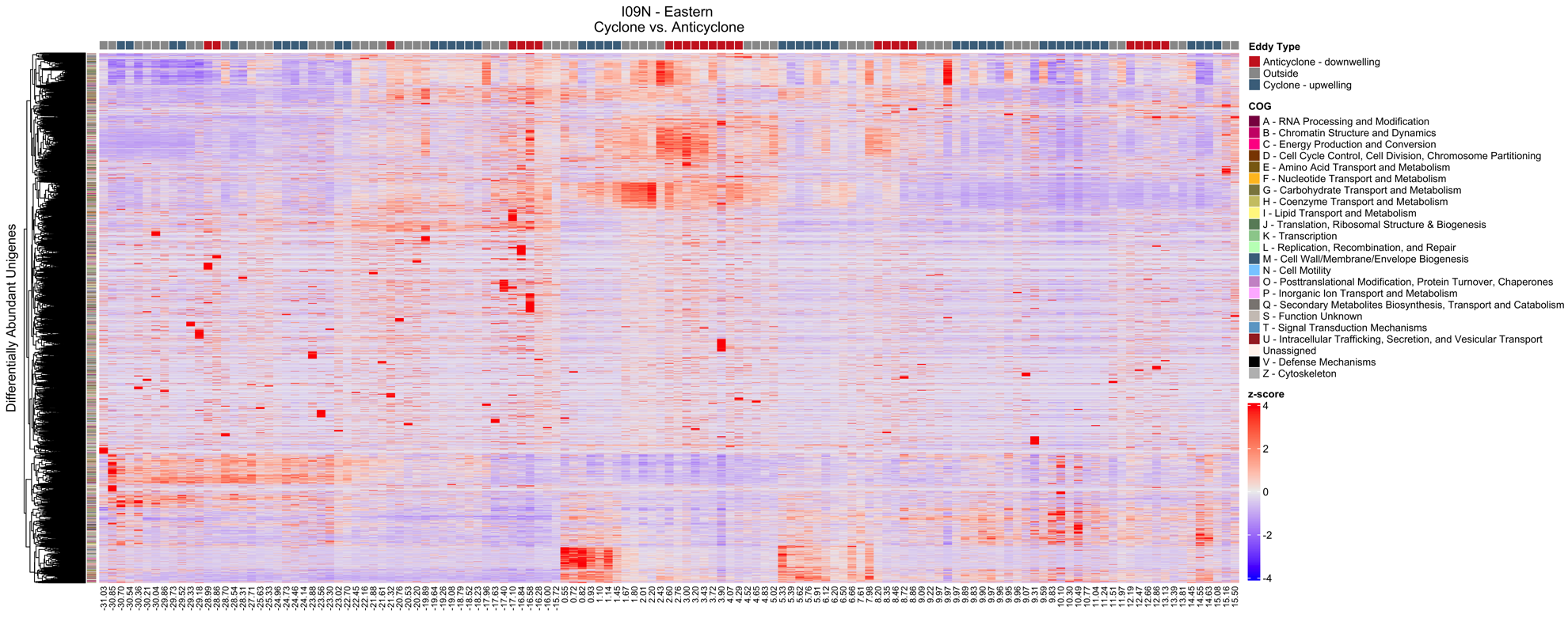
**

**Supplemental Figure 16: Heatmap of differentially abundant unigenes found in a pairwise comparison of eddy types in the eastern Indian Ocean.** Each row represents a unigene, and each column represents a sample labeled by its latitude. Unigenes are color-scaled according to their z-scores to highlight relative increases or decreases. The dendrogram clusters unigenes based on their normalized, scaled abundances to aid in identifying patterns across samples. Colored boxes above the heatmap indicate the eddy type associated with each sample, while colored boxes on the side denote the COG category assigned to each unigene.
