# Supplementary material for: Roles of physical disturbance and biome properties in shaping microbial communities within Indian Ocean eddies
Source: ISME Commun. 2025 Jul 2;5(1):ycaf110. doi: 10.1093/ismeco/ycaf110 (PMC12306440; doi:10.1093/ismeco/ycaf110)
Supplement: MLB_IndianOceanEddies_ISME_Supplemental_Methods_wRef_ycaf110 [file mlb_indianoceaneddies_isme_supplemental_methods_wref_ycaf110.docx]

**Supplemental Materials and Methods**

*Field Sampling and Environmental Data*

Microbial DNA and environmental metrics were collected on two GO-SHIP transects, I09N and I07N, in the Indian Ocean. GO-SHIP samples are collected at predefined stations along repeat hydrographic sections to support sustained oceanographic observations. In the eastern Indian Ocean, samples (n = 215) were collected during the intermonsoon season in 2016 (22 March–24 April) on GO-SHIP cruise I09N, which ran from Freemont, Australia to Phuket, Thailand. In the western Indian Ocean, samples (n = 250) were collected during the intermonsoon season in 2018 (23 April–06 June) on GO-SHIP cruise I07N which ran from Durban, South Africa to Mormugao, India.

For microbial DNA samples, surface seawater was collected every 4–6 h from the ship's circulating seawater system at 7 m depth (n = 414) or via Niskin rosette at 3 m depth (n = 51). Between 1 and 10 L of surface seawater was filtered through a 0.22 μm Sterivex filter. Filters were preserved with 1620 μL of lysis buffer (23.4 mg mL^−1^ NaCl, 257 mg mL^−1^ sucrose, 50 mmol L^−1^ Tris–HCl, 20 mmol L^−1^ EDTA). Filters were then frozen at −20°C until further processing.

On both cruises, temperature and nutrient concentrations were measured through the following protocols. Underway temperature was measured continuously using a mounted near-surface thermosalinograph. Samples for inorganic nutrient concentrations of nitrate, nitrite, phosphate, and silicate were collected at pre-defined GO-SHIP stations at approximately every latitudinal degree (~ 11 km) for the entire water column using a Niskin rosette. All nutrient concentrations were measured following standard GO-SHIP protocols [1] on a Seal Analytical continuous-flow AutoAnalyzer3 using a 10 mm flowcell. For nitrate and nitrite concentrations, a modification of the 1967 Armstrong procedure [2] was performed. For nitrate analysis, seawater was passed through a copper-cadmium reduction column where nitrate was reduced to nitrite. Nitrite was diazotized with sulfanilamide and coupled with N-1-naphthyl-ethylenediamine dihydrochloride (N-1-N/NEDD), resulting in a red azo dye. Sample absorbance was measured at 540 nm. The same procedure but without using the cadmium column was repeated for nitrite analysis. For ortho-phosphate concentrations, a modification of the 1967 Bernhardt and Wilhelms procedure [3] was performed. Phosphomolybdic acid was produced by adding acidified ammonium molybdate to seawater samples. The phosphomolybdic acid was then reduced to a phospho-molybdenum blue complex after the addition of dihydrazine sulfate. Sample absorbance was measured at either 820 or 880 nm. For silicate concentrations, the 1967 method of Armstrong et al. [2] was performed. Silicomolybdic acid was produced by adding ammonium molybdate to seawater. The silicomolybdic acid was reduced to form a silico-molybdenum complex following the addition of stannous chloride. Sample absorbance was measured at 660 nm. For underway sampling points between GO-SHIP stations, nutrient concentrations were linearly interpolated from the nutrient concentrations of the nearest GO-SHIP stations.

Nutricline depth was used as a proxy for nutrient supply to the mixed layer [4] and was defined as the depth at which nitrate was ≥ 1 μmol L^−1^. To estimate the nutricline depth at each GO-SHIP station, nitrate depth profiles were interpolated at 1 m resolution. At underway sampling points between GO-SHIP stations, the nutricline depth was interpolated from the nutricline depths of the nearest GO-SHIP stations. For underway samples collected before the first GO-SHIP station, World Ocean Atlas climatological nitrate depth profiles were used to estimate the nutricline depth.

*DNA Extraction*

Microbial DNA was extracted from the Sterivex filters. Filters were thawed and incubated with lysozyme (50 mg mL^−1^ final concentration) for 30 min at 37°C. Samples were then incubated at 55°C overnight with proteinase K (1 mg mL^−1^) and 10% SDS buffer. DNA was precipitated using sodium acetate (245 mg mL^−1^, pH 5.2) and ice-cold isopropanol (100%). DNA was pelleted via centrifugation at 15,000 × g at 4°C for 30 min. The pelleted DNA was resuspended in TE buffer (10 mmol L^−1^ Tris–HCl, 1 mmol L^−1^ EDTA) at 37°C for 1 h and was then purified and concentrated using a Zymo genomic DNA Clean and Concentrator kit (Zymo Research Corp.). DNA concentration was measured using a Qubit dsDNA HS Assay on a Qubit fluorometer (Thermo Fisher Scientific).

*16S rRNA Amplification and Sequencing*

The 515F-C and 926R primer set [5] was used to amplify the V4-V5 region of the 16S rRNA gene in all samples collected on the eastern (I09N) and western (I07N) transects. DNA samples were diluted to 2 ng μL^−1^. A total of 4 μL of diluted DNA was added to 20 μL reactions containing 0.4 μmol L^−1^ of each primer and 1X AccuStart II PCR Supermix (final concentration). Two polymerase chain reactions (PCRs) were performed. The first PCR was performed as follows: 1 cycle of 94°C for 3 min and 26 cycles of 94°C for 30 s, 55°C for 30 s, and 68°C for 40 s. Free primers and primer dimers were removed from PCR products using a magnetic bead cleanup containing 10 μL of Milli-Q water, 10 μL of PCR product, and 20 μL of Sera-mag SpeedBeads. Barcodes were annealed to the bead-cleaned products in a second PCR. A total of 4 μL of bead-cleaned PCR product was added to 20 μL reactions containing 0.3 μmol L^−1^ of an i5 Nextera v2 index, 0.3 μmol L^−1^ of an i7 Nextera v2 index, and 1X AccuStart II PCR Supermix (final concentration). The second PCR was performed as follows: 12 cycles of 94°C for 30 s, 55°C for 30 s, 68°C for 40 s; and a final extension of 68°C for 10 min. Final PCR products were visualized with a 1% agarose gel, and products were pooled based on band brightness. Unincorporated barcodes were removed from the pooled library with a final magnetic bead cleanup that contained 60 μL of pooled product and 60 μL of Sera-mag SpeedBeads. DNA concentration of the library was assessed using a Qubit dsDNA HS Assay on a Qubit fluorometer (Thermo Fisher Scientific), and library quality was assessed using a Bioanalyzer (Agilent). The amplicon library was pair-end sequenced (2 × 300 bp) on an Illumina MiSeq platform. Sequence files are available under BioProject ID PRJNA656268 at the NCBI Sequence Read Archive (SRA).

*rpoC1 Amplification and Sequencing*

Cyanobacteria-specific primers 5M_newF (5′-GARCARATHGTYTAYTTYA-3′) and SAC1039R (5′-CYTGYTTNCCYTCDATDATRT-3′) [6] with Illumina-specific Nextera transposase adapters were used to amplify the *rpo*C1 gene in all samples collected on the eastern transect (I09N). DNA samples were diluted to 1 ng μL^−1^. A total of 2 μL of the diluted DNA was added to 20 μL reactions containing 0.3 μmol L^−1^ of each primer, 2.5 units of 5Prime HotMaster DNA Taq Polymerase, and MasterAmp 1× Premix F. A two-step PCR was performed. The first PCR proceeded as follows: 1 cycle of 94°C for 2 min and 34 cycles of 94°C 30 s, 48°C 40 s, and 72°C 60 s. Next, 1 μL each of i5 and i7 Nextera v2 indices (1 ng μL^−1^) were added to the products and were PCR annealed to the amplicons in a second PCR which proceeded as follows: 10 cycles of 94°C 30 s, 55°C 40 s, and 72°C 60 s; and a final extension step at 72°C for 10 min. Products were pooled and dimers < 700 bp in length were removed using a bead cleanup with Agencourt AMPure XP beads. A 2100 Bioanalyzer high sensitivity DNA trace (Agilent) was used to assess dimer removal. The amplicon library was pair-end sequenced (2 × 300 bp) on an Illumina MiSeq platform with 600 cycles. Sequence files are available under BioProject ID PRJNA522445 at the NCBI SRA.

*Short-read Metagenome Library Preparation and Sequencing*

Illumina-specific Nextera DNA transposase adapters and an Illumina Tagment DNA Enzyme and Buffer Kit were used to prepare metagenomic libraries from all samples collected on the eastern (I09N) and western (I07N) transect. DNA samples were diluted to 2 ng μL^−1^. A total of 1 μL of the diluted DNA was added to 1.5 μL tagmentation reactions containing 1.25 μL of TD buffer and 0.25 μL of TDE1. Tagmentation reactions were incubated at 55 °C for 10 minutes. Following tagmentation, 2.5 μL of product was immediately added to 22 μL reactions to anneal barcodes to the tagmentation products. Custom Nextera DNA-style 8 bp unique dual index (UDI) barcodes were used: i7 (5′-CAA GCA GAA GAC GGC ATA CGA GAT [NNN NNN NN]G TCT CGT GGG CTC GG-3′) and i5 (5′-AAT GAT ACG GCG ACC ACC GAG ATC TAC AC[N NNN NNN N]TC GTC GGC AGC GTC-3′). For bioinformatic quality trimming, the Nextera adapter sequences to be used are: 5′-TCG TCG GCA GCG TCA GAT GTG TAT AAG AGA CAG-3′ and 5′-GTC TCG TGG GCT CGG AGA TGT GTA TAA GAG ACA G-3′. In addition to tagmented product, the 22 μL reactions contained 1.02 μM of UDI barcode, 204 μM of dNTPs, 0.0204 U of Phusion High Fidelity DNA polymerase, and 1.02X Phusion HF Buffer (final concentration). Barcodes were annealed with the following PCR: 1 cycle of 72 °C for 2 min, 1 cycle of 98 °C for 30 s, 13 cycles of 98 °C 10 s, 63 °C 30 s, and 72 °C 30 s; and a final extension step of 72 °C for 5 min. Dimers that were less than 150 nucleotides long were removed from tagmentation products using a buffered solution of Sera-mag SpeedBeads containing 1 M NaCl, 1 mM EDTA, 10 mM Tris-HCl, 44.4 M PEG-8000, and 0.055% Tween-20 (final concentrations). DNA concentrations of metagenomic libraries were quantified using a Qubit dsDNA HS Assay kit and a Synergy 2 Microplate Reader (BioTek, Winooski, VT). Metagenomic libraries were pooled at equimolar concentrations, and a KAPA qPCR platform was used to verify the pooled library concentration. Dimer removal and read size distribution were assessed using a 2100 Bioanalyzer high sensitivity DNA trace (Agilent). The majority of the samples from I09N and all samples from I07N were sequenced on Illumina NovaSeq S4 flowcells using 150 bp paired-end chemistry with 300 cycles. An additional 24 samples from I09N were sequenced on an Illumina HiSeq 4000 lane using 150 bp paired-end chemistry with 300 cycles. Of these 24 samples, 8 of them were identified as eddy samples or non-eddy control samples and were included in subsequent analyses. Sequence files are available under BioProject ID PRJNA656268 at the NCBI SRA.

*Bioinformatics for 16S rRNA Amplicons*

Bioinformatics methods for 16S rRNA amplicons are summarized in Figure 1 of the Supplemental Materials and Methods. Cutadapt [7] implemented in QIIME2 [8] was used to remove primers from reads. Fastq-mcf [9] was used to quality filter forward and reverse reads. Mean quality score was calculated using a window size of 10. When the mean quality score was less than 20, reads were trimmed at that position. After trimming, reads shorter than a minimum length threshold of 150 bp were removed. Any reads containing N-calls were also removed. Usearch [10] was used to merge forward and reverse reads based on the following parameters: a minimum overlap threshold of 10 bp, a minimum merge length threshold of 350 bp, and a maximum of 5 bp differences allowed in the overlapping region. DADA2 [11] implemented in QIIME2 [8] was used to perform final trimming, quality filtering, clustering of amplicons, and removal of chimeras. To maintain alignment, the merged reads were trimmed to a length threshold of 304 bp. Reads matching to the PhiX genome were removed. An error model was trained using a minimum of 800,000 reads, and reads that contained more than 3 expected errors were removed. Samples were then dereplicated, and reads were clustered into amplicon sequence variants (ASVs). Chimeric ASVs were removed using a consensus procedure. To minimize the effect that rare ASVs resulting from differences in sequencing runs may have on diversity metrics, the remaining ASVs were clustered into de novo 99% operational taxonomic units (OTUs) using the VSEARCH plugin [12] implemented in QIIME2 [8].

*Bioinformatics for rpoC1 Amplicons*

Bioinformatics methods for *rpo*C1 amplicons are summarized in Figure 1 of the Supplemental Materials and Methods. FASTQC was used to truncate the forward read to 294 bp and the reverse read to 222 bp to maintain an average quality score >20. Due to the length of the *rpo*C1 gene, forward and reverse reads could not be merged. Instead, the reads were concatenated to a total length of 516 bp. Fastq-mcf [9] was used to remove reads with a mean quality score below 20 and to remove reads with more than 1% of bases below Q20. 97% OTUs were grouped using the pick_open_reference_otus.py function in QIIME [13] with a custom database of *Synechococcus* and *Prochlorococcus* reference sequences in order to identify *Prochlorococcus* sequences. Reference sequences were trimmed to the same length as query sequences. The reference database contained 15 *Synechococcus* and 41 *Prochlorococcus* fully sequenced genomes [14] as well as 2 metagenomic assemblies, one for eHL-III, which was 97.8% complete, and one for eHL-IV, which was 97.1% complete [15]. The 97% OTU representative sequences were taxonomically identified using blastn (version 2.6.0, e-value < 10−5, identity > 80%) with comparison to the NCBI Prokaryotic RefSeq genomes database. Any sequences with < 80% identity to *Prochlorococcus* were removed. To identify *Prochlorococcus* ecotypes, reads were then re-compared using blastn (e-value < 10−5, identity > 90%) to the custom hand-curated and annotated database of *Synechococcus* and *Prochlorococcus* sequences.


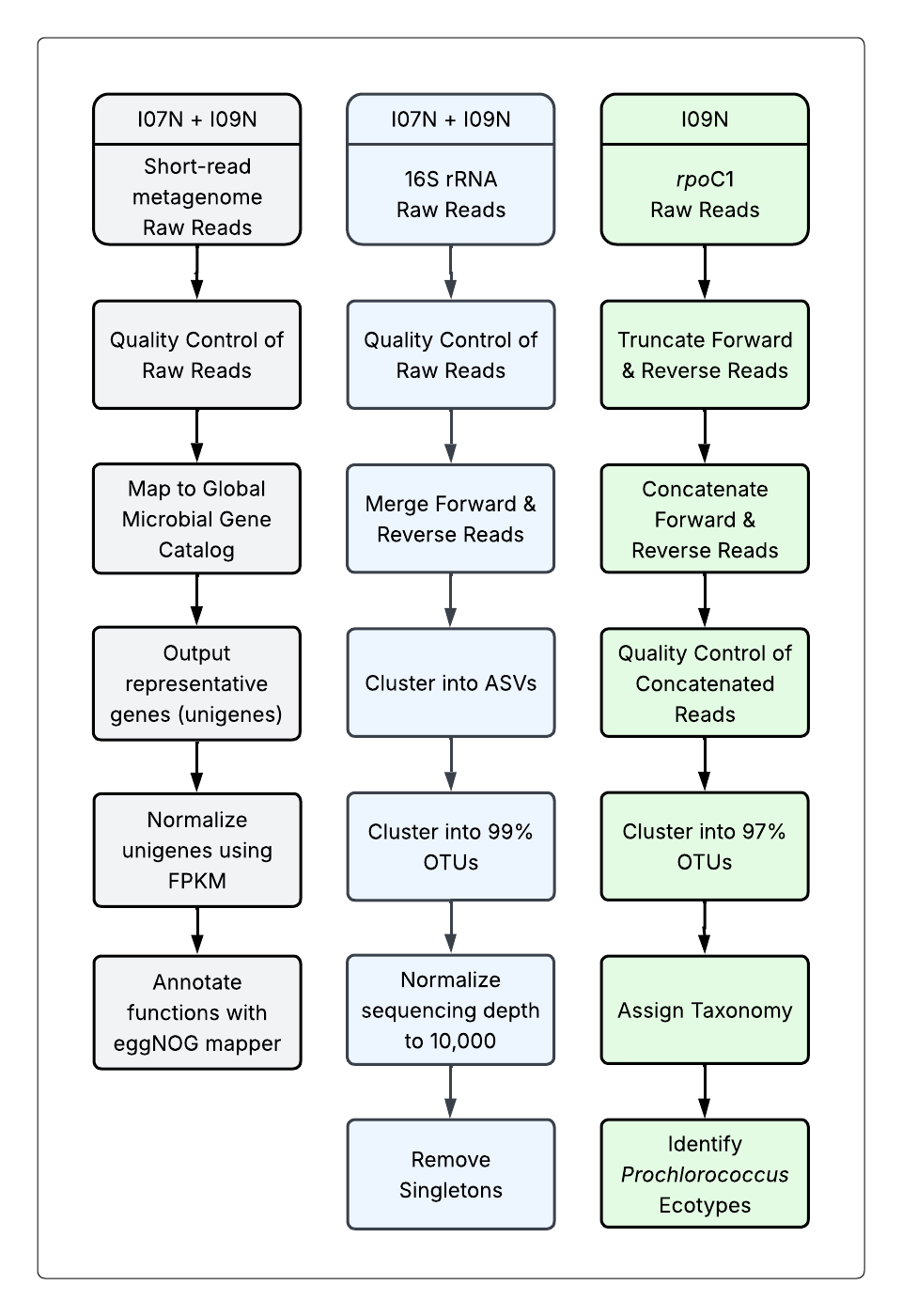


**Figure 1: Flowchart of bioinformatics methods for different sequence types.** Cruise identifiers in the top boxes denote which cruises each type of sequencing was performed for. I07N = the western Indian Ocean transect and I09N = the eastern Indian Ocean transect.

**References**

1. Becker S, Aoyama M, Woodward EMS, Bakker K, Coverly S, Mahaffey C, et al. GO-SHIP Repeat Hydrography Nutrient Manual: The Precise and Accurate Determination of Dissolved Inorganic Nutrients in Seawater, Using Continuous Flow Analysis Methods. Front Mar Sci. 2020 Oct 30;7.

2. Wood ED, Armstrong FAJ, Richards FA. Determination of nitrate in sea water by cadmium-copper reduction to nitrite. Journal of the Marine Biological Association of the United Kingdom. 1967 Feb 11;47(1):23–31.

3. Bernhardt H, Wilhelms A. The continuous determination of low level iron, soluble phosphate and total phosphate with the AutoAnalyzer. Technicon symposia. 1967;1:385–9.

4. Cermeño P, Dutkiewicz S, Harris RP, Follows M, Schofield O, Falkowski PG. The role of nutricline depth in regulating the ocean carbon cycle. Proc Natl Acad Sci U S A. 2008 Dec 23;105(51):20344–9.

5. Needham DM, Fuhrman JA. Pronounced daily succession of phytoplankton, archaea and bacteria following a spring bloom. Nat Microbiol. 2016 Feb 29;1(4):16005.

6. Kent AG, Baer SE, Mouginot C, Huang JS, Larkin AA, Lomas MW, et al. Parallel phylogeography of Prochlorococcus and Synechococcus. ISME J. 2019 Feb 1;13(2):430–41.

7. Martin M. Cutadapt removes adapter sequences from high-throughput sequencing reads. EMBnet J. 2011 May 2;17(1):10.

8. Bolyen E, Rideout JR, Dillon MR, Bokulich NA, Abnet CC, Al-Ghalith GA, et al. Reproducible, interactive, scalable and extensible microbiome data science using QIIME 2. Nat Biotechnol. 2019 Aug 1;37(8):852–7.

9. Aronesty E. Comparison of sequencing utility programs. Open Bioinforma J. 2013 Feb 1;7(1):1–8.

10. Edgar RC. Search and clustering orders of magnitude faster than BLAST. Bioinformatics. 2010 Oct 1;26(19):2460–1.

11. Callahan BJ, McMurdie PJ, Rosen MJ, Han AW, Johnson AJA, Holmes SP. DADA2: High-resolution sample inference from Illumina amplicon data. Nature Methods 2016 13:7. 2016 May 23;13(7):581–3.

12. Rognes T, Flouri T, Nichols B, Quince C, Mahé F. VSEARCH: A versatile open source tool for metagenomics. PeerJ. 2016 Oct 18;2016(10):e2584.

13. Caporaso JG, Kuczynski J, Stombaugh J, Bittinger K, Bushman FD, Costello EK, et al. QIIME allows analysis of high-throughput community sequencing data. Nat Methods. 2010 May 11;7(5):335–6.

14. Biller SJ, Berube PM, Berta-Thompson JW, Kelly L, Roggensack SE, Awad L, et al. Genomes of diverse isolates of the marine cyanobacterium Prochlorococcus. Sci Data. 2014 Sep 30;1(1):140034.

15. Kent AG, Dupont CL, Yooseph S, Martiny AC. Global biogeography of Prochlorococcus genome diversity in the surface ocean. ISME Journal. 2016 Aug 1;10(8):1856–65.
